# Crystal structure of tris­[bis­(2,6-diiso­propyl­phen­yl) phosphato-κ*O*]penta­kis­(methanol-κ*O*)europium methanol monosolvate

**DOI:** 10.1107/S2056989019015421

**Published:** 2019-11-19

**Authors:** Alexey E. Kalugin, Konstantin A. Lyssenko, Mikhail E. Minyaev, Dmitrii M. Roitershtein, Lada N. Puntus, Evgenia A. Varaksina, Ilya E. Nifant’ev

**Affiliations:** a A.V. Topchiev Institute of Petrochemical Synthesis, Russian Academy of Sciences, 29 Leninsky Prospect, Moscow, 119991, Russian Federation; b Moscow Institute of Physics and Technology, Department of Biological and Medical Physics, 9 Institutskiy Per., Dolgoprudny, Moscow Region, 141701, Russian Federation; c G.V. Plekhanov Russian University of Economics, 36, Stremyanny Per., Moscow, 117997, Russian Federation; d Chemistry Department, M.V. Lomonosov Moscow State University, 1 Leninskie Gory, Building 3, Moscow, 119991, Russian Federation; e N.D. Zelinsky Institute of Organic Chemistry, Russian Academy of Sciences, 47 Leninsky Prospect, Moscow, 119991, Russian Federation; f V.A. Kotel’nikov Institute of Radioengineering and Electronics, Russian Academy of Sciences, 11-7 Mokhovaya Str., Moscow, 125009, Russian Federation; g P.N. Lebedev Physical Institute, Russian Academy of Sciences, 53 Leninsky Prospect, Moscow 119991, Russian Federation

**Keywords:** crystal structure, europium, organophosphate, coordination compound, isostructural, hydrogen bonding, luminescence

## Abstract

The mononuclear title complex, [Eu(C_24_H_34_O_4_P)_3_(CH_4_O)_5_]·CH_4_O, (**1**), has been obtained as a minor product in the reaction between EuCl_3_(H_2_O)_6_ and lithium bis­(2,6-diiso­propyl­phen­yl) phosphate in a 1:3 molar ratio in a methanol medium. Its structure exhibits monoclinic (*P*2_1_/*c*) symmetry at 120 K and is isostructural with the La, Ce and Nd analogs reported previously [Minyaev *et al.* (2018*a* ▸). *Acta Cryst.* C**74**, 590–598]. In (**1**), all three bis­(2,6-diiso­propyl­phen­yl) phosphate ligands display the terminal κ^1^ *O*-coordination mode. All of the hy­droxy H atoms are involved in O—H⋯O hydrogen bonding, exhibiting four intra­molecular and two inter­molecular hydrogen bonds. Photophysical studies have demonstrated luminescence of (**1**) with a low quantum yield.

## Abstract 2

The crystal structure of the complex {Eu[O_2_P(O-2,6-^*i*^Pr_2_C_6_H_3_)_2_]_3_(CH_3_OH)_5_}·CH_3_OH, which exhibits intra- and inter­molecular O—H⋯O hydrogen bonding, and its luminescent properties have been studied.

## Chemical context   

Rare-earth complexes with organic ligands are widely used as reagents, catalysts or precatalysts in organic synthesis or in various polymerization reactions and even in technological processes. For example, complexes with organophosphate ligands are used in the polymerization of 1,3-dienes (Anwander, 2002 ▸; Friebe *et al.*, 2006 ▸; Kobayashi & Anwander, 2001 ▸; Minyaev *et al.*, 2018*a* ▸,*b* ▸,*c* ▸; Nifant’ev *et al.*, 2013 ▸, 2014 ▸; Zhang *et al.*, 2010 ▸). Rare-earth organophosphates are also formed during the isolation and separation of lanthanides in industry (Atwood, 2016 ▸; Chen, 2016 ▸).

The luminescence of coordination compounds of certain lanthanide cations (Eu^3+^, Tb^3+^, Dy^3+^, Nd^3+^ *etc*.) is well-known (Bünzli, 2017 ▸); however, the photophysical properties of rare-earth organophosphates have not been reported so far. Meanwhile, a so-called ‘antenna’ ligand possessing a conjugated π-electron system may increase the quantum yield of lanthanide complexes dramatically (Bünzli & Piguet, 2005 ▸; Guillou *et al.*, 2016 ▸). In order to examine the possibility of applying a disubstituted organophosphate anion as an ‘antenna’ ligand for luminescence sensitization, we have chosen the bis­(2,6-diiso­propyl­phen­yl) phosphate anion, which allows single crystals of mono- and binuclear rare-earth complexes to be obtained (Minyaev *et al.*, 2017 ▸, 2018*a* ▸,*b* ▸), unlike most other di(alk­yl/ar­yl) phosphate ligands that do not provide crystallizable lanthanide compounds. Mononuclear rare-earth com­plexes with this ligand form two isotructural series of bis- and tris­(phosphate) complexes: {*Ln*[O_2_P(O-2,6-^*i*^Pr_2_C_6_H_3_)_2_]_2_Cl(CH_3_OH)_4_}·2CH_3_OH (*Ln* = Nd, Y, Lu; Minyaev *et al.*, 2017 ▸) and {*Ln*[O_2_P(O-2,6-^*i*^Pr_2_C_6_H_3_)_2_]_3_(CH_3_OH)_5_}·CH_3_OH (*Ln* = La, Ce, Nd; Minyaev *et al.*, 2018*a* ▸). It was found that the bis­(phosphate) monochloride complex of Nd is thermally unstable in a solution and can be easily converted into the corresponding tris­(phosphate) complex upon mild heating (>310 K) in methanol. Moreover, bis(phosphate) monochloride complexes of lighter lanthanides cannot be obtained. However, the heaviest lanthanide for obtaining the tris(phosphate) complex has not been determined. Herein, we report on the crystal structure and luminescent properties of the complex {Eu[O_2_P(O-2,6-^*i*^Pr_2_C_6_H_3_)_2_]_3_(CH_3_OH)_5_}·CH_3_OH (**1**), which bears the heaviest lanthanide within the tris­(phosphate) series (Minyaev *et al.*, 2018*a* ▸)
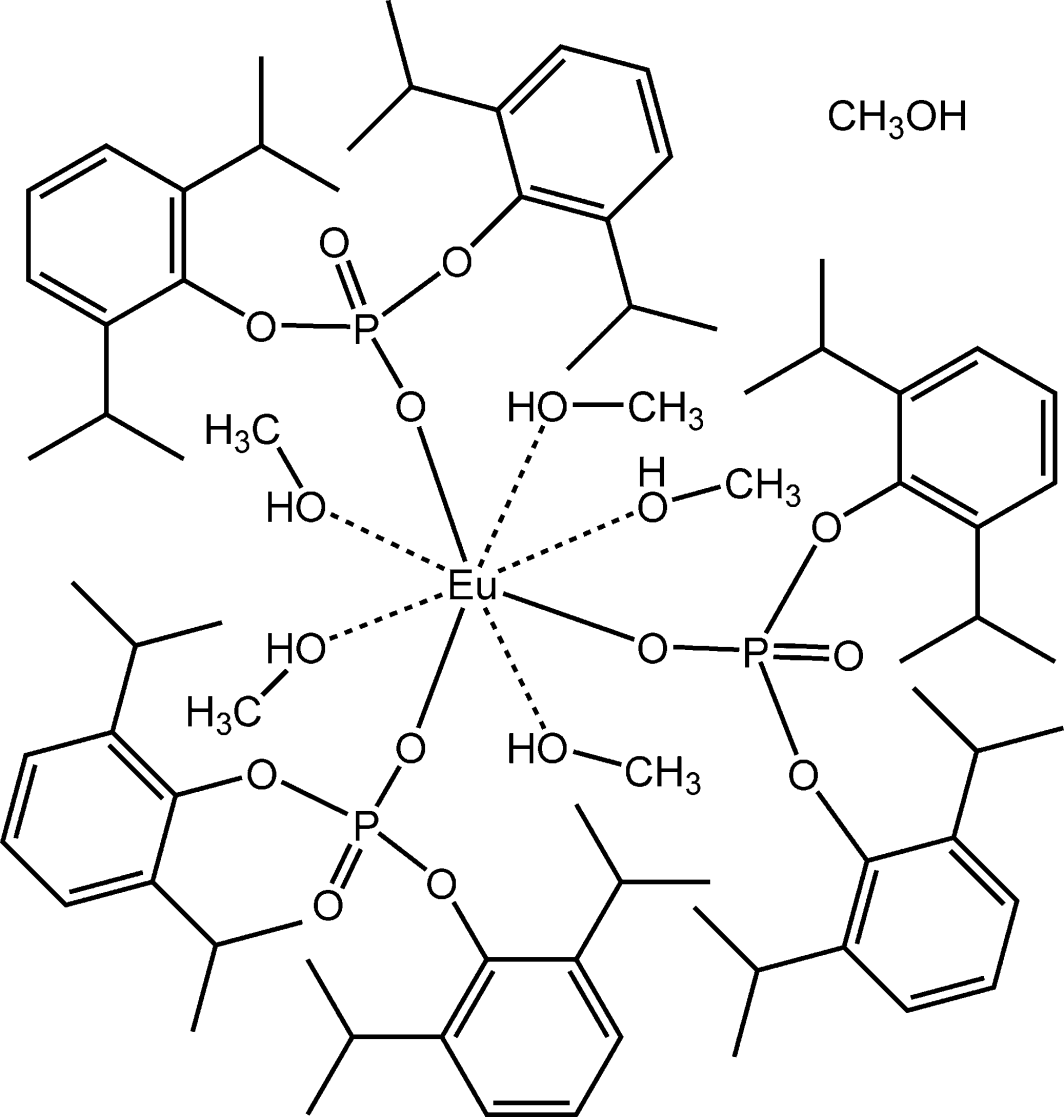
.

## Structural commentary   

**Figure 1:**
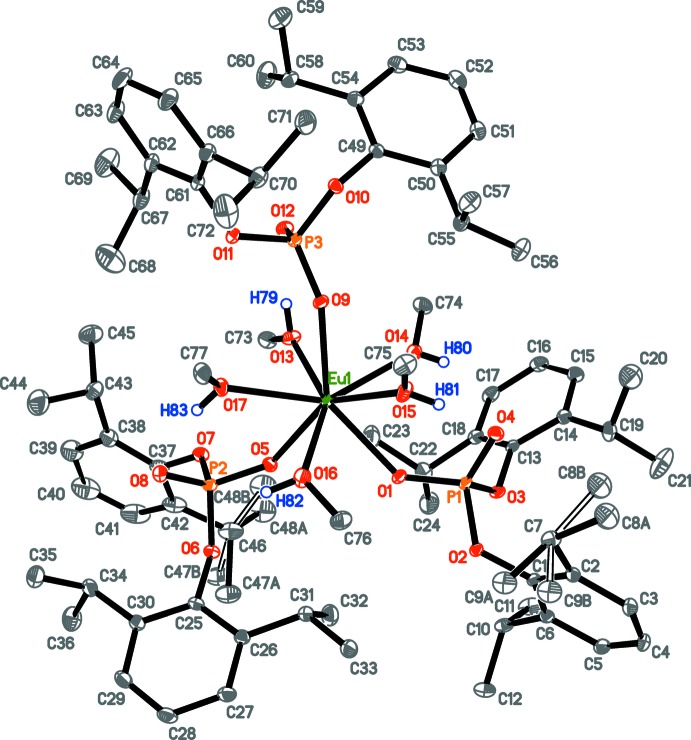
Mol­ecular structure of complex (**1**), with atom labelling. Displacement ellipsoids are drawn at the 30% probability level. For clarity, the solvent methanol mol­ecule and the C-bound H atoms have been omitted. Minor components of the disordered isopropyl group are shown with open solid lines.

The asymmetric unit of (**1**) contains the complex [Eu{O_2_P(O-2,6-^*i*^Pr_2_C_6_H_3_)_2_}_2_(CH_3_OH)_5_] and one non-coordinating methanol mol­ecule (Fig. 1 ▸). Selected bond distances in complex (**1**) are given in Table 1 ▸. The Eu^3+^ cation is coordinated by five methanol mol­ecules and three di­aryl­phosphate ligands displaying the terminal κ^1^ *O*-coordination mode, which leads to the Eu^3+^ coordination number of eight. Two phosphate ligands are located close to each other (atoms P1, P2), but the third phosphate ligand (atom P3) is separated from them by the methanol mol­ecules. The complex itself does not have any symmetry element (the *C*_1_ point group), but in a rough approximation, the EuO_8_ core might be thought of as belonging to the *C*_s_ point group with a mirror plane passing through atoms Eu1, O9 and O16. This supports the conclusions drawn from photophysical studies about the Eu^3+^ environment (see §4).

The Eu—O_P_ distances are on average 0.11 Å shorter than Eu—O_MeOH_ (Table 1 ▸), being in agreement with ion–ion and ion–dipole *Ln*–ligand inter­action types, accordingly. The phospho­rous atoms are in a distorted tetra­hedral environment. The smallest O—P—O bond angle in each ligand corresponds to the O_C_—P—O_C_ angle between bulky aryl substituents [99.08 (8)° for O2—P1—O3; 100.80 (9)° for O6—P2—O7, 101.24 (8)° for O10—P3—O11], whereas the largest bond angles are for O_*Ln*_—P=O [114.89 (9)° for O1—P1—O4, 116.23 (9)° for O5—P2—O8, 116.11 (9)° for O9—P3—O12]. The O—C_*ipso*_ bond lengths [1.402 (3)–1.413 (2) Å; Table 1 ▸] are only slightly shorter (by ∼0.02 Å) than a regular single O—C bond length. The P—O_*Ln*_ and P=O bond lengths are nearly identical and on average 0.10 Å shorter than the P—O_C_ distances. The values of P—O bonds and O—P—O angles indicate a more pronounced double-bond character for the P—O_*Ln*_ and P=O bonds with nearly equal charge redistribution on the two corresponding oxygen atoms (Minyaev *et al.*, 2017 ▸). A roughly single-bond character for both the O—C_*ipso*_ and P—O_C_ bonds indicates no conjugation between the aryl fragments and the phosphorus atom and consequently prevents charge transfer from aryl groups to Eu^3+^. Therefore, the chosen organophosphate is inapplicable as an ‘antenna’ ligand, which is in agreement with the rather low quantum yield of the complex (see §4).

## Supra­molecular features   

**Figure 2:**
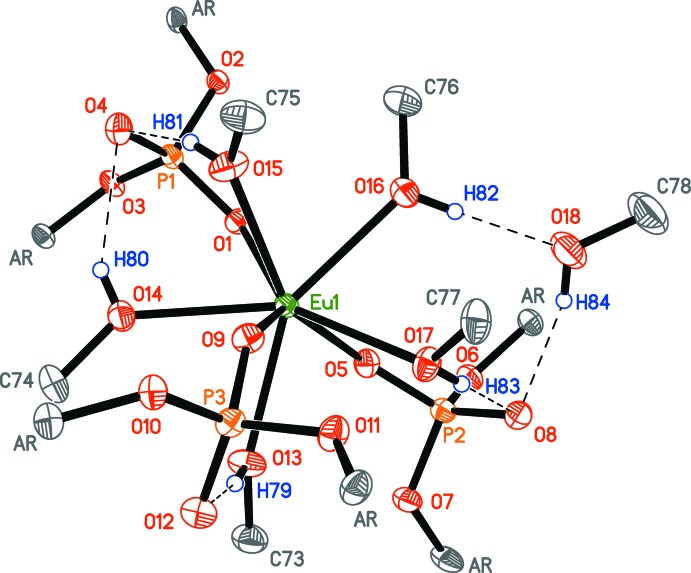
Intra- and inter­molecular O—H⋯O bonding in the crystal structure of complex (**1**). Only core atoms and hy­droxy H atoms are shown. Atomic displacement parameters are set to the 50% probability level.

Complex (**1**) forms four intra­molecular O—H⋯O hydrogen bonds and two inter­molecular hydrogen bonds with one non-coordinating methanol mol­ecule, yielding a mol­ecular associate {[(O_2_P(OAr)_2_)_3_Eu(MeOH)_5_]·MeOH} (Fig. 2 ▸, Table 2 ▸). The presence of the two-dimensional hydrogen-bonding network in bis­(diaryl phosphate) complexes [*Ln*(O_2_P(OAr)_2_)_2_Cl(CH_3_OH)_4_]·2CH_3_OH (Minyaev *et al.*, 2017 ▸) substanti­ally decreases their solubility compared to tris­(diaryl phosphate) complexes [*Ln*(O_2_P(OAr)_2_)_3_(CH_3_OH)_5_]·CH_3_OH, which do not have such a network, and which are soluble in aromatic and aliphatic hydro­carbons (Minyaev *et al.*, 2018*a* ▸). Likely due to both this fact and incomplete reaction, the precipitate contains complex (**2**) as a major product (see §5, Fig. 4 ▸), which is isostructural to the bis­(diaryl phosphate) monochloride complexes.

## Luminescence studies   

**Figure 3:**
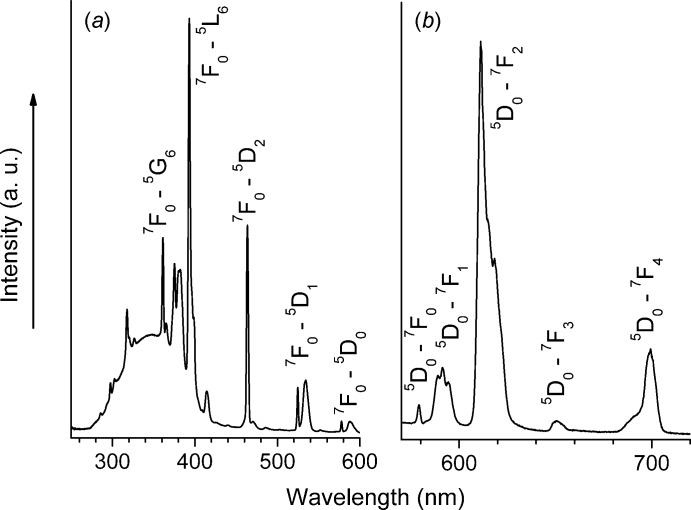
Luminescence excitation spectrum (*a*), and luminescence spectrum (*b*), of complex (**1**) at 300 K.

The steady-state luminescence excitation spectrum of (**1**) (Fig. 3 ▸ *a*) was recorded in the spectroscopic range from 250 to 600 nm with emission monitored on the hypersensitive ^5^ *D*_0_→^7^ *F*_2_ transition at 612 nm. This spectrum consists of narrow bands assigned to the 4*f*–4*f* intra­configurational transitions and a broad band centered around 350 nm. The latter could be tentatively assigned to an inter­ligand charge-transfer (ILCT) band due to the presence of the anion-assisted strong hydrogen bonding between coordinated methanol mol­ecules and oxygen atoms at the O=P bonds of the organophosphate ligands (see §3 and Fig. 2 ▸). A similar charge-transfer band was observed in the case of lanthanide triflates, where the charge redistribution caused by inter­molecular hydrogen bonds resulting in an additional CT state was found and confirmed by combined research of luminescence data and the experimental electron density distribution function analysis (Nelyubina *et al.*, 2014 ▸).

The emission spectrum of (**1**) (Fig. 3 ▸ *b*), recorded in the range from 400 to 720 nm under excitation at 394 nm (^7^ *F*_0_→^5^ *L*_6_ transition), exhibits intense narrow bands corres­ponding to the ^5^ *D*_0_→^7^ *F_J_* transitions (*J* = 0–4). These electronic transitions display the maximum possible number of Stark components pointing to a low site symmetry for Eu^3+^, *i.e.* equal to or lower than *C*_2*v*_. Generally, the intensities and Stark splittings of the ^5^ *D*_0_→^7^ *F_J_* transitions are influenced by the strength and symmetry of the ligand. A forbidden ^5^ *D*_0_→^7^ *F*_0_ transition (region 570–585 nm) of the Eu^3+^ cation is presented by a relatively intense symmetric line that indicates the presence of only one type of Eu environment. The integrated intensity of this transition is 0.13, which corresponds to a relatively strong deviation of the Eu^3+^ site symmetry from *C_i_*. The electric dipole ^5^ *D*_0_→^7^ *F*_2_ transition (region 600–620 nm) is extremely sensitive to the symmetry of the europium surroundings and called *hypersensitive*, and so the ratio of integrated intensities of the ^5^ *D*_0_→^7^ *F*_2_ transition to ^5^ *D*_0_→^7^ *F*_1_ is a measure of the symmetry of the coordination sphere. In a centrosymmetric environment the magnetic dipole ^5^ *D*_0_→^7^ *F*_1_ transition is dominating and the above ratio is < 1, while the distortion of the symmetry around the ion causes an intensity enhancement of the ^5^ *D*_0_→^7^ *F*_2_ transition. In (**1**), this ratio equals 5, which points to a remarkable deviation from a centrosymmetric environment of the Eu^3+^ ion. These facts correlate with the found site symmetry for Eu^3+^ from the X-ray data (see Figs. 1 ▸ and 2 ▸). The high intensity of the first Stark component of the ^5^ *D*_0_→^7^ *F*_2_ transition at 300 K can potentially be used for obtaining a relatively high colour purity (the line at 610 nm, ∼50% of the total integrated intensity). Furthermore, a weak broad band was observed in this spectrum in the region 400–550 nm, indicating the residual luminescence of the ligands. Consequently, the overall quantum yield is quite low for the complex (∼2.5%), which prevents the use of complex (**1**) in luminescent applications.

## Synthesis   

**Figure 4:**
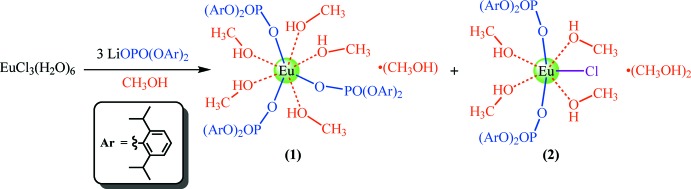
Synthesis of {Eu[O_2_P(O-2,6-^*i*^Pr_2_C_6_H_3_)_2_]_3_(CH_3_OH)_5_}·CH_3_OH, (**1**), and {Eu[O_2_P(O-2,6-^*i*^Pr_2_C_6_H_3_)_2_]_2_Cl(CH_3_OH)_4_}·CH_3_OH (**2**).

Complex (**1**) was obtained as a minor product in the reaction of lithium bis­(2,6-diiso­propyl­phen­yl) phosphate with EuCl_3_(H_2_O)_6_ in a 3:1 ratio in methanol at room temperature (Fig. 4 ▸). Only a few single crystal samples were represented by analytically pure (**1**), whereas the precipitated bulk microcrystalline product was a mixture and mainly contained {Eu[O_2_P(O-2,6-^*i*^Pr_2_C_6_H_3_)_2_]_2_Cl(CH_3_OH)_4_}·CH_3_OH (**2**), according to IR and C/H analysis. The structure and photophysical properties of (**2**) will be reported elsewhere. Attempts to isolate (**1**) as the only product in this reaction failed. Furthermore, attempts to synthesize and grow single crystals of the analogous Tb and Gd tris­(phosphate) complexes failed as well. Therefore, the isostructural complexes {*Ln*[O_2_P(O-2,6-^i^Pr_2_C_6_H_3_)_2_]_3_(CH_3_OH)_5_}·CH_3_OH can only be obtained for lanthanides from La to Eu.

### General experimental remarks   

The synthesis of (**1**) was carried out under an argon atmosphere. Methanol was distilled over Ca/Mg alloy and stored over mol­ecular sieves (4 Å). The salt [{(2,6-^*i*^Pr_2_C_6_H_3_-O)_2_POO}Li(MeOH)_3_]·MeOH was prepared according to the literature (Minyaev *et al.*, 2015 ▸). C/H elemental analysis was performed with a PerkinElmer 2400 Series II elemental analyser. Steady-state luminescence and excitation measurements in the visible region were performed with a Fluoro­log FL 3-22 spectrometer from Horiba–Jobin–Yvon–Spex, which has a 450 W xenon lamp as the excitation source and an R-928 photomultiplier. The quantum yield measurements were carried out on solid samples with a Spectralone-covered G8 integration sphere (GMP SA, Switzerland) under ligand excitation, according to the absolute method by Wrighton (Wrighton *et al.*, 1974 ▸; de Mello *et al.*, 1997 ▸; Greenham *et al.*, 1995 ▸).

### Synthetic procedure   

A solution of [{(2,6-^*i*^Pr_2_C_6_H_3_-O)_2_POO}Li(MeOH)_3_]·MeOH (3.315 g, 6.00 mmol) in methanol (12 ml) was added to a stirred solution of EuCl_3_·6H_2_O (0.733 g, 2.00 mmol) in methanol (5 ml). Then, the reaction mixture was allowed to stand overnight at room temperature. Some single crystals (∼150 mg) that had formed on the walls of the flask were taken for X-ray studies and elemental analysis, which showed that their composition corresponds to (**1**). Analysis found (calculated for C_78_H_126_EuO_18_P_3_) (%): C 58.79 (58.67), H 8.02 (7.95).

The remaining reaction mixture was kept at room temperature for 2 days and for 1 day in a freezer (255 K). The formed precipitate was filtered off, washed with cold (268 K) methanol (3 × 5 ml), then dried under vacuum to provide 1.861 g of a microcrystalline product. The C/H elemental analysis and FT IR studies demonstrated that the formed product contains (**2**) with some impurities of (**1**) and possibly of the starting lithium salt.

Numerous attempts to obtain (**1**) as a single product by varying the reaction conditions failed.

## Refinement   

Crystal data, data collection and structure refinement details are summarized in Table 3 ▸. The positions of all non-H and hy­droxy H atoms were found from difference electron-density maps. All other H atoms were also found from difference-Fourier maps (with the exception of the disordered fragments) but were positioned geometrically (C—H = 0.95 Å for aromatic, 0.98 Å for methyl, 1.00 Å for tertiary hydrogen atoms) and refined as riding atoms with *U*_iso_(H) = 1.5*U*_eq_(C-meth­yl) and 1.2*U*_eq_(C) for other H atoms. A rotating group model was applied for the methyl groups. Reflection 100 was affected by the beam stop, and omitted from the final refinement. Atoms C8, C9 and C47, C48 and corresponding H atoms were disordered over two positions in two isopropyl fragments. Since the residual electron density was not enough to properly position minor components of the disordered isopropyl groups, initial positions for corresponding carbon atoms were taken from isostructural compounds (Minyaev *et al.*, 2018*a* ▸). This allowed the disorder to be resolved successfully [the disorder ratios are 0.921 (5):0.079 (5) for atoms C8*A*, C9*A* / C8*B*, C9*B* and 0.879 (6):0.121 (6) for C47*A*, C48*A* / C47*B*, C48*B*] and to improve the crystallographic model slightly.

## Supplementary Material

Crystal structure: contains datablock(s) I, global. DOI: 10.1107/S2056989019015421/su5526sup1.cif

Structure factors: contains datablock(s) I. DOI: 10.1107/S2056989019015421/su5526Isup2.hkl

Supporting information file. DOI: 10.1107/S2056989019015421/su5526Isup3.cdx

CCDC references: 1965700, 1965700

Additional supporting information:  crystallographic information; 3D view; checkCIF report

## Figures and Tables

**Table 1:** Selected bond lengths (Å)

Eu1—O1	2.3915 (14)	P2—O7	1.5978 (16)
Eu1—O5	2.3166 (15)	P2—O8	1.4923 (17)
Eu1—O9	2.3525 (15)	P3—O9	1.5010 (16)
Eu1—O13	2.4374 (16)	P3—O10	1.6007 (16)
Eu1—O14	2.4933 (16)	P3—O11	1.5970 (16)
Eu1—O15	2.4312 (17)	P3—O12	1.4855 (17)
Eu1—O16	2.4664 (17)	O2—C1	1.413 (2)
Eu1—O17	2.4665 (16)	O3—C13	1.413 (2)
P1—O1	1.4963 (16)	O6—C25	1.410 (3)
P1—O2	1.5991 (15)	O7—C37	1.402 (3)
P1—O3	1.5935 (16)	O10—C49	1.411 (3)
P1—O4	1.4922 (16)	O11—C61	1.406 (3)
P2—O5	1.4972 (16)	O13—C73	1.420 (3)
P2—O6	1.5938 (16)		

**Table 2:** Hydrogen-bond geometry (Å, °)

*D*—H⋯*A*	*D*—H	H⋯*A*	*D*⋯*A*	*D*—H⋯*A*
O13—H79⋯O12	0.81 (3)	1.83 (3)	2.632 (2)	171 (3)
O14—H80⋯O4	0.76 (3)	2.27 (3)	2.941 (2)	148 (3)
O15—H81⋯O4	0.82 (3)	1.79 (3)	2.583 (2)	160 (3)
O16—H82⋯O18	0.82 (3)	1.86 (3)	2.684 (3)	178 (4)
O17—H83⋯O8	0.82 (3)	1.99 (3)	2.783 (2)	165 (3)
O18—H84⋯O8	0.82 (4)	1.94 (4)	2.723 (3)	160 (4)

**Table 3:** Experimental details

Crystal data	
Chemical formula	[Eu(C_24_H_34_O_4_P)_3_(CH_4_O)_5_]·CH_4_O
*M*_r_	1596.65
Crystal system, space group	Monoclinic, *P*2_1_/*c*
Temperature (K)	120
*a*, *b*, *c* (Å)	23.4010 (17), 10.6604 (8), 33.543 (2)
β (°)	91.964 (1)
*V* (Å^3^)	8363.0 (11)
*Z*	4
Radiation type	Mo *K*α
μ (mm^−1^)	0.87
Crystal size (mm)	0.46 × 0.36 × 0.22

Data collection	
Diffractometer	Bruker APEXII CCD area-detector
Absorption correction	Multi-scan (*SADABS*; Krause *et al.*, 2015 ▸)
*T*_min_, *T*_max_	0.644, 0.748
No. of measured, independent and observed [*I* > 2σ(*I*)] reflections	158417, 29661, 24028
*R*_int_	0.061
(sin θ/λ)_max_ (Å^−1^)	0.752

Refinement	
*R*[*F* ^2^ > 2σ(*F* ^2^)], *wR*(*F* ^2^), *S*	0.049, 0.091, 1.16
No. of reflections	29661
No. of parameters	973
No. of restraints	12
H-atom treatment	H atoms treated by a mixture of independent and constrained refinement
Δρ_max_, Δρ_min_ (e Å^−3^)	1.27, −1.33

Computer programs: *APEX2* and *SAINT* (Bruker, 2016 ▸), *SHELXT2013* (Sheldrick, 2015*a* ▸), *SHELXL2018* (Sheldrick, 2015*b* ▸) and *SHELXTL* (Sheldrick, 2008 ▸).

## supplementary crystallographic information

### Crystal data

**Table:**

[Eu(C_24_H_34_O_4_P)_3_(CH_4_O)_5_]·CH_4_O	*F*(000) = 3384
*M**_r_* = 1596.65	*D*_x_ = 1.268 Mg m^−^^3^
Monoclinic, *P*2_1_/*c*	Mo *K*α radiation, λ = 0.71073 Å
*a* = 23.4010 (17) Å	Cell parameters from 9998 reflections
*b* = 10.6604 (8) Å	θ = 2.5–30.2°
*c* = 33.543 (2) Å	µ = 0.87 mm^−^^1^
β = 91.964 (1)°	*T* = 120 K
*V* = 8363.0 (11) Å^3^	Block, colorless
*Z* = 4	0.46 × 0.36 × 0.22 mm

### Data collection

**Table:**

Bruker APEXII CCD area-detector diffractometer	24028 reflections with *I* > 2σ(*I*)
Radiation source: X-Ray tube	*R*_int_ = 0.061
ω scans	θ_max_ = 32.3°, θ_min_ = 1.5°
Absorption correction: multi-scan (SADABS; Krause *et al.*, 2015)	*h* = −35→35
*T*_min_ = 0.644, *T*_max_ = 0.748	*k* = −15→16
158417 measured reflections	*l* = −50→50
29661 independent reflections	

### Refinement

**Table:**

Refinement on *F*^2^	Primary atom site location: structure-invariant direct methods
Least-squares matrix: full	Secondary atom site location: difference Fourier map
*R*[*F*^2^ > 2σ(*F*^2^)] = 0.049	Hydrogen site location: mixed
*wR*(*F*^2^) = 0.091	H atoms treated by a mixture of independent and constrained refinement
*S* = 1.16	*w* = 1/[σ^2^(*F*_o_^2^) + (0.024*P*)^2^ + 8.2744*P*] where *P* = (*F*_o_^2^ + 2*F*_c_^2^)/3
29661 reflections	(Δ/σ)_max_ = 0.001
973 parameters	Δρ_max_ = 1.27 e Å^−^^3^
12 restraints	Δρ_min_ = −1.33 e Å^−^^3^

### Special details

**Table:**

Experimental. moisture sensitive
Geometry. All esds (except the esd in the dihedral angle between two l.s. planes) are estimated using the full covariance matrix. The cell esds are taken into account individually in the estimation of esds in distances, angles and torsion angles; correlations between esds in cell parameters are only used when they are defined by crystal symmetry. An approximate (isotropic) treatment of cell esds is used for estimating esds involving l.s. planes.

### Fractional atomic coordinates and isotropic or equivalent isotropic displacement parameters (Å^2^)

**Table:**

	*x*	*y*	*z*	*U*_iso_*/*U*_eq_	Occ. (<1)
Eu1	0.21264 (2)	0.01198 (2)	0.12253 (2)	0.01371 (3)	
P1	0.30150 (2)	0.10405 (5)	0.04210 (2)	0.01430 (10)	
P2	0.28809 (2)	0.07752 (5)	0.21773 (2)	0.01634 (10)	
P3	0.09000 (2)	−0.19974 (5)	0.12637 (2)	0.01590 (10)	
O1	0.29035 (6)	0.09169 (14)	0.08558 (4)	0.0172 (3)	
O2	0.32035 (6)	0.24664 (13)	0.03573 (4)	0.0150 (3)	
O3	0.35910 (6)	0.03625 (13)	0.03007 (5)	0.0174 (3)	
O4	0.25347 (7)	0.06299 (15)	0.01474 (5)	0.0196 (3)	
O5	0.27848 (7)	0.05181 (14)	0.17413 (5)	0.0187 (3)	
O6	0.34194 (7)	0.16726 (14)	0.22491 (5)	0.0185 (3)	
O7	0.31298 (7)	−0.05192 (14)	0.23493 (5)	0.0204 (3)	
O8	0.23761 (7)	0.12372 (15)	0.23943 (5)	0.0203 (3)	
O9	0.12428 (6)	−0.09030 (14)	0.11230 (5)	0.0180 (3)	
O10	0.04631 (6)	−0.24582 (14)	0.09163 (5)	0.0177 (3)	
O11	0.04729 (7)	−0.13794 (15)	0.15688 (5)	0.0193 (3)	
O12	0.12327 (7)	−0.30716 (15)	0.14321 (5)	0.0208 (3)	
O13	0.22177 (7)	−0.19159 (15)	0.15521 (5)	0.0223 (3)	
O14	0.23584 (8)	−0.14526 (16)	0.07049 (5)	0.0233 (4)	
O15	0.16784 (7)	0.08781 (17)	0.06066 (5)	0.0241 (4)	
O16	0.19455 (8)	0.23884 (16)	0.12912 (6)	0.0267 (4)	
O17	0.15557 (7)	0.05522 (16)	0.18145 (5)	0.0213 (3)	
O18	0.19450 (10)	0.33364 (19)	0.20326 (7)	0.0414 (5)	
C1	0.34644 (9)	0.29122 (18)	0.00110 (6)	0.0156 (4)	
C2	0.31138 (10)	0.3397 (2)	−0.02967 (7)	0.0192 (4)	
C3	0.33846 (12)	0.3867 (2)	−0.06304 (7)	0.0267 (5)	
H3A	0.315991	0.418677	−0.084867	0.032*	
C4	0.39734 (12)	0.3873 (2)	−0.06481 (7)	0.0294 (6)	
H4A	0.414962	0.418716	−0.087872	0.035*	
C5	0.43060 (11)	0.3426 (2)	−0.03324 (7)	0.0245 (5)	
H5A	0.471046	0.344730	−0.034773	0.029*	
C6	0.40629 (10)	0.29440 (19)	0.00091 (7)	0.0181 (4)	
C7	0.24736 (10)	0.3513 (2)	−0.02465 (6)	0.0234 (5)	
H7A	0.234295	0.274598	−0.010405	0.028*	0.921 (5)
H7B	0.236648	0.316383	0.001739	0.028*	0.079 (5)
C8A	0.21244 (15)	0.3613 (4)	−0.06395 (10)	0.0450 (9)	0.921 (5)
H8A	0.171582	0.361802	−0.058434	0.067*	0.921 (5)
H8B	0.220955	0.289401	−0.080955	0.067*	0.921 (5)
H8C	0.222413	0.439127	−0.077620	0.067*	0.921 (5)
C9A	0.23577 (12)	0.4649 (3)	0.00187 (9)	0.0316 (7)	0.921 (5)
H9A	0.194886	0.468914	0.007185	0.047*	0.921 (5)
H9B	0.247245	0.541763	−0.011750	0.047*	0.921 (5)
H9C	0.257749	0.456734	0.027131	0.047*	0.921 (5)
C8B	0.2173 (17)	0.278 (4)	−0.0586 (9)	0.0450 (9)	0.079 (5)
H8D	0.232223	0.192191	−0.059081	0.067*	0.079 (5)
H8E	0.224418	0.319236	−0.084093	0.067*	0.079 (5)
H8F	0.176099	0.275951	−0.054395	0.067*	0.079 (5)
C9B	0.2356 (14)	0.4926 (7)	−0.0262 (12)	0.0316 (7)	0.079 (5)
H9D	0.258671	0.534868	−0.005344	0.047*	0.079 (5)
H9E	0.194950	0.507916	−0.022001	0.047*	0.079 (5)
H9F	0.245597	0.525343	−0.052389	0.047*	0.079 (5)
C10	0.44439 (10)	0.2534 (2)	0.03586 (8)	0.0216 (5)	
H10A	0.419456	0.220624	0.057124	0.026*	
C11	0.48529 (11)	0.1481 (2)	0.02417 (10)	0.0350 (6)	
H11A	0.463186	0.077680	0.012902	0.052*	
H11B	0.507338	0.119821	0.047856	0.052*	
H11C	0.511455	0.179541	0.004278	0.052*	
C12	0.47853 (11)	0.3653 (2)	0.05312 (8)	0.0298 (5)	
H12A	0.452034	0.431120	0.061125	0.045*	
H12B	0.503655	0.398296	0.032831	0.045*	
H12C	0.501645	0.337737	0.076415	0.045*	
C13	0.36357 (9)	−0.09326 (18)	0.02182 (6)	0.0146 (4)	
C14	0.34882 (10)	−0.1371 (2)	−0.01624 (7)	0.0201 (4)	
C15	0.35576 (11)	−0.2660 (2)	−0.02265 (8)	0.0257 (5)	
H15A	0.345594	−0.300033	−0.048100	0.031*	
C16	0.37686 (11)	−0.3449 (2)	0.00683 (8)	0.0265 (5)	
H16A	0.380607	−0.432115	0.001734	0.032*	
C17	0.39259 (11)	−0.2967 (2)	0.04389 (8)	0.0247 (5)	
H17A	0.407541	−0.351437	0.064021	0.030*	
C18	0.38688 (10)	−0.1687 (2)	0.05227 (7)	0.0200 (4)	
C19	0.32908 (11)	−0.0507 (2)	−0.05019 (7)	0.0254 (5)	
H19A	0.321176	0.033530	−0.038465	0.030*	
C20	0.27450 (14)	−0.0958 (3)	−0.07127 (10)	0.0480 (8)	
H20A	0.244699	−0.106829	−0.051735	0.072*	
H20B	0.281637	−0.176113	−0.084439	0.072*	
H20C	0.261889	−0.033768	−0.091241	0.072*	
C21	0.37619 (14)	−0.0344 (4)	−0.08014 (11)	0.0576 (10)	
H21A	0.362362	0.019521	−0.102075	0.086*	
H21B	0.386736	−0.116601	−0.090728	0.086*	
H21C	0.409721	0.004249	−0.066849	0.086*	
C22	0.40669 (11)	−0.1158 (2)	0.09240 (7)	0.0275 (5)	
H22A	0.399249	−0.023411	0.091847	0.033*	
C23	0.37436 (13)	−0.1700 (4)	0.12613 (9)	0.0441 (8)	
H23A	0.333258	−0.157875	0.120958	0.066*	
H23B	0.386026	−0.127742	0.151045	0.066*	
H23C	0.382642	−0.259877	0.128444	0.066*	
C24	0.47118 (13)	−0.1346 (4)	0.09959 (10)	0.0496 (9)	
H24A	0.491544	−0.102417	0.076679	0.074*	
H24B	0.479369	−0.224221	0.102951	0.074*	
H24C	0.483845	−0.089261	0.123716	0.074*	
C25	0.34322 (9)	0.2981 (2)	0.23117 (7)	0.0181 (4)	
C26	0.35429 (10)	0.3743 (2)	0.19848 (7)	0.0199 (4)	
C27	0.36167 (11)	0.5027 (2)	0.20599 (7)	0.0259 (5)	
H27A	0.369503	0.557474	0.184521	0.031*	
C28	0.35777 (12)	0.5514 (2)	0.24417 (8)	0.0295 (5)	
H28A	0.363051	0.638639	0.248684	0.035*	
C29	0.34625 (11)	0.4730 (2)	0.27547 (8)	0.0273 (5)	
H29A	0.343127	0.507456	0.301423	0.033*	
C30	0.33900 (10)	0.3437 (2)	0.27000 (7)	0.0212 (4)	
C31	0.35890 (11)	0.3194 (2)	0.15686 (7)	0.0231 (5)	
H31A	0.326026	0.260526	0.152359	0.028*	
C32	0.41402 (12)	0.2435 (3)	0.15305 (8)	0.0344 (6)	
H32A	0.416355	0.179608	0.174051	0.052*	
H32B	0.447060	0.299681	0.155769	0.052*	
H32C	0.413903	0.202586	0.126877	0.052*	
C33	0.35514 (13)	0.4191 (2)	0.12401 (8)	0.0314 (6)	
H33A	0.321475	0.471960	0.127758	0.047*	
H33B	0.351866	0.378012	0.097895	0.047*	
H33C	0.389656	0.471204	0.125346	0.047*	
C34	0.32933 (11)	0.2598 (2)	0.30559 (7)	0.0270 (5)	
H34A	0.321392	0.173134	0.295437	0.032*	
C35	0.27772 (13)	0.3025 (3)	0.32878 (9)	0.0392 (7)	
H35A	0.243589	0.302799	0.311015	0.059*	
H35B	0.284572	0.387325	0.339163	0.059*	
H35C	0.271911	0.244849	0.351025	0.059*	
C36	0.38329 (14)	0.2545 (3)	0.33275 (9)	0.0412 (7)	
H36A	0.415176	0.221621	0.317693	0.062*	
H36B	0.376530	0.199468	0.355507	0.062*	
H36C	0.392678	0.339039	0.342406	0.062*	
C37	0.33968 (11)	−0.0723 (2)	0.27244 (8)	0.0242 (5)	
C38	0.30635 (12)	−0.1100 (2)	0.30395 (8)	0.0290 (5)	
C39	0.33523 (16)	−0.1341 (3)	0.34042 (9)	0.0471 (8)	
H39A	0.314020	−0.157803	0.362860	0.057*	
C40	0.39399 (17)	−0.1242 (3)	0.34430 (11)	0.0591 (11)	
H40A	0.412792	−0.139637	0.369374	0.071*	
C41	0.42525 (15)	−0.0920 (3)	0.31199 (11)	0.0507 (9)	
H41A	0.465699	−0.087238	0.315048	0.061*	
C42	0.39955 (12)	−0.0660 (2)	0.27489 (9)	0.0343 (6)	
C43	0.24237 (12)	−0.1318 (2)	0.29975 (8)	0.0302 (6)	
H43A	0.229910	−0.109187	0.271861	0.036*	
C44	0.20934 (17)	−0.0495 (4)	0.32799 (11)	0.0567 (9)	
H44A	0.218863	0.038782	0.323421	0.085*	
H44B	0.219665	−0.071998	0.355632	0.085*	
H44C	0.168213	−0.062118	0.323107	0.085*	
C45	0.22876 (14)	−0.2708 (3)	0.30595 (10)	0.0425 (7)	
H45A	0.249024	−0.321265	0.286510	0.064*	
H45B	0.187490	−0.284262	0.302278	0.064*	
H45C	0.241064	−0.295838	0.333011	0.064*	
C46	0.43517 (12)	−0.0380 (2)	0.23901 (10)	0.0384 (7)	
H46A	0.407885	−0.018698	0.216239	0.046*	0.879 (6)
H46B	0.419015	0.028504	0.220861	0.046*	0.121 (6)
C47A	0.47333 (15)	0.0771 (3)	0.24513 (13)	0.0456 (10)	0.879 (6)
H47A	0.449611	0.150298	0.250796	0.068*	0.879 (6)
H47B	0.494513	0.092490	0.220909	0.068*	0.879 (6)
H47C	0.500346	0.062332	0.267608	0.068*	0.879 (6)
C48A	0.4709 (2)	−0.1503 (4)	0.22652 (15)	0.0563 (12)	0.879 (6)
H48A	0.445744	−0.222125	0.220990	0.084*	0.879 (6)
H48B	0.498479	−0.171761	0.248099	0.084*	0.879 (6)
H48C	0.491452	−0.129025	0.202459	0.084*	0.879 (6)
C47B	0.4921 (6)	−0.004 (3)	0.2600 (8)	0.0456 (10)	0.121 (6)
H47D	0.485387	0.056385	0.281429	0.068*	0.121 (6)
H47E	0.517573	0.033975	0.240704	0.068*	0.121 (6)
H47F	0.509973	−0.079572	0.271277	0.068*	0.121 (6)
C48B	0.4407 (15)	−0.1670 (16)	0.2197 (10)	0.0563 (12)	0.121 (6)
H48D	0.404101	−0.190405	0.206717	0.084*	0.121 (6)
H48E	0.451078	−0.229054	0.240283	0.084*	0.121 (6)
H48F	0.470388	−0.164233	0.199873	0.084*	0.121 (6)
C49	0.05436 (9)	−0.3542 (2)	0.06837 (7)	0.0173 (4)	
C50	0.07807 (9)	−0.3385 (2)	0.03096 (7)	0.0188 (4)	
C51	0.08245 (10)	−0.4440 (2)	0.00685 (7)	0.0221 (5)	
H51A	0.098632	−0.436287	−0.018625	0.027*	
C52	0.06351 (10)	−0.5603 (2)	0.01950 (7)	0.0247 (5)	
H52A	0.066756	−0.631541	0.002718	0.030*	
C53	0.03996 (10)	−0.5723 (2)	0.05648 (7)	0.0241 (5)	
H53A	0.027024	−0.652320	0.064815	0.029*	
C54	0.03473 (9)	−0.4696 (2)	0.08188 (7)	0.0197 (4)	
C55	0.09369 (10)	−0.2085 (2)	0.01617 (7)	0.0212 (4)	
H55A	0.109703	−0.159638	0.039417	0.025*	
C56	0.13787 (11)	−0.2079 (3)	−0.01627 (8)	0.0313 (6)	
H56A	0.170977	−0.258446	−0.007578	0.047*	
H56B	0.150160	−0.121489	−0.021151	0.047*	
H56C	0.120854	−0.243198	−0.040892	0.047*	
C57	0.03877 (11)	−0.1430 (2)	0.00102 (8)	0.0294 (5)	
H57A	0.011304	−0.140367	0.022410	0.044*	
H57B	0.022173	−0.189466	−0.021774	0.044*	
H57C	0.047745	−0.057267	−0.007266	0.044*	
C58	0.00699 (11)	−0.4858 (2)	0.12172 (7)	0.0260 (5)	
H58A	0.010071	−0.403990	0.136218	0.031*	
C59	−0.05638 (13)	−0.5171 (3)	0.11623 (10)	0.0445 (7)	
H59A	−0.075631	−0.450622	0.100700	0.067*	
H59B	−0.073516	−0.523716	0.142396	0.067*	
H59C	−0.060728	−0.597104	0.102049	0.067*	
C60	0.03792 (15)	−0.5845 (3)	0.14730 (9)	0.0459 (8)	
H60A	0.078558	−0.562892	0.150145	0.069*	
H60B	0.033802	−0.666761	0.134490	0.069*	
H60C	0.021272	−0.587219	0.173697	0.069*	
C61	−0.00403 (9)	−0.1868 (2)	0.17102 (7)	0.0190 (4)	
C62	−0.00188 (10)	−0.2613 (2)	0.20546 (7)	0.0244 (5)	
C63	−0.05394 (12)	−0.3024 (3)	0.21908 (8)	0.0324 (6)	
H63A	−0.054265	−0.355201	0.241895	0.039*	
C64	−0.10539 (12)	−0.2691 (3)	0.20049 (8)	0.0355 (6)	
H64A	−0.140382	−0.298664	0.210554	0.043*	
C65	−0.10568 (11)	−0.1927 (3)	0.16726 (8)	0.0304 (6)	
H65A	−0.141150	−0.169203	0.154814	0.036*	
C66	−0.05483 (10)	−0.1494 (2)	0.15159 (7)	0.0224 (5)	
C67	0.05420 (11)	−0.2904 (3)	0.22732 (8)	0.0302 (6)	
H67A	0.082275	−0.312903	0.206676	0.036*	
C68	0.07755 (17)	−0.1752 (4)	0.24915 (11)	0.0607 (10)	
H68A	0.079783	−0.105043	0.230402	0.091*	
H68B	0.115802	−0.193621	0.260471	0.091*	
H68C	0.052119	−0.152853	0.270647	0.091*	
C69	0.05114 (15)	−0.4018 (3)	0.25585 (10)	0.0514 (9)	
H69A	0.037031	−0.475715	0.241205	0.077*	
H69B	0.025090	−0.381868	0.277214	0.077*	
H69C	0.089345	−0.419170	0.267412	0.077*	
C70	−0.05634 (11)	−0.0629 (2)	0.11569 (7)	0.0257 (5)	
H70A	−0.016147	−0.052738	0.107013	0.031*	
C71	−0.09102 (14)	−0.1184 (3)	0.08078 (8)	0.0409 (7)	
H71A	−0.075503	−0.200865	0.074078	0.061*	
H71B	−0.088917	−0.062655	0.057653	0.061*	
H71C	−0.130983	−0.127515	0.088128	0.061*	
C72	−0.07845 (17)	0.0672 (3)	0.12657 (10)	0.0525 (9)	
H72A	−0.053461	0.103669	0.147551	0.079*	
H72B	−0.117383	0.059910	0.136202	0.079*	
H72C	−0.078686	0.121267	0.102952	0.079*	
C73	0.25778 (11)	−0.2619 (2)	0.18182 (8)	0.0272 (5)	
H73A	0.263511	−0.345980	0.170877	0.041*	
H73B	0.239879	−0.268570	0.207721	0.041*	
H73C	0.294793	−0.219504	0.185220	0.041*	
C74	0.23787 (11)	−0.2793 (2)	0.06745 (8)	0.0276 (5)	
H74A	0.259497	−0.303013	0.044102	0.041*	
H74B	0.198881	−0.312447	0.064585	0.041*	
H74C	0.256615	−0.314088	0.091570	0.041*	
C75	0.11132 (11)	0.1241 (2)	0.04977 (8)	0.0299 (6)	
H75A	0.107720	0.133813	0.020744	0.045*	
H75B	0.102512	0.204044	0.062629	0.045*	
H75C	0.084557	0.059678	0.058404	0.045*	
C76	0.20148 (13)	0.3414 (2)	0.10225 (8)	0.0320 (6)	
H76A	0.223493	0.408173	0.115746	0.048*	
H76B	0.163821	0.373748	0.093691	0.048*	
H76C	0.221872	0.312691	0.078909	0.048*	
C77	0.10100 (11)	0.1178 (3)	0.17943 (8)	0.0293 (5)	
H77A	0.073545	0.070603	0.194968	0.044*	
H77B	0.087151	0.122754	0.151573	0.044*	
H77C	0.105163	0.202673	0.190388	0.044*	
C78	0.19089 (16)	0.4513 (3)	0.22041 (11)	0.0472 (8)	
H78A	0.169256	0.445689	0.244852	0.057*	
H78B	0.171403	0.508764	0.201636	0.057*	
H78C	0.229450	0.482716	0.226892	0.057*	
H79	0.1921 (14)	−0.231 (3)	0.1539 (10)	0.045 (10)*	
H80	0.2377 (13)	−0.114 (3)	0.0501 (9)	0.036 (9)*	
H81	0.1898 (13)	0.088 (3)	0.0420 (9)	0.037 (9)*	
H82	0.1952 (14)	0.267 (3)	0.1521 (10)	0.044 (10)*	
H83	0.1746 (13)	0.075 (3)	0.2013 (9)	0.032 (8)*	
H84	0.2103 (15)	0.283 (3)	0.2184 (11)	0.052 (11)*	

### Atomic displacement parameters (Å^2^)

**Table:**

	*U*^11^	*U*^22^	*U*^33^	*U*^12^	*U*^13^	*U*^23^
Eu1	0.01357 (5)	0.01276 (4)	0.01492 (5)	0.00006 (4)	0.00238 (3)	0.00046 (4)
P1	0.0142 (2)	0.0120 (2)	0.0169 (3)	−0.00063 (18)	0.0042 (2)	−0.00030 (19)
P2	0.0168 (3)	0.0148 (2)	0.0173 (3)	−0.00023 (19)	−0.0006 (2)	0.0004 (2)
P3	0.0133 (2)	0.0182 (2)	0.0163 (3)	−0.00221 (19)	0.0019 (2)	0.0004 (2)
O1	0.0163 (7)	0.0174 (7)	0.0181 (7)	−0.0021 (6)	0.0047 (6)	0.0023 (6)
O2	0.0167 (7)	0.0123 (6)	0.0162 (7)	−0.0002 (5)	0.0055 (6)	−0.0002 (5)
O3	0.0178 (7)	0.0108 (6)	0.0239 (8)	−0.0004 (5)	0.0063 (6)	−0.0006 (6)
O4	0.0173 (7)	0.0204 (7)	0.0211 (8)	−0.0030 (6)	0.0018 (6)	−0.0035 (6)
O5	0.0188 (8)	0.0183 (7)	0.0188 (8)	−0.0007 (6)	0.0006 (6)	−0.0020 (6)
O6	0.0189 (8)	0.0146 (7)	0.0217 (8)	−0.0010 (6)	−0.0016 (6)	−0.0012 (6)
O7	0.0228 (8)	0.0156 (7)	0.0226 (8)	0.0009 (6)	−0.0024 (6)	0.0022 (6)
O8	0.0218 (8)	0.0208 (7)	0.0185 (8)	0.0016 (6)	0.0013 (6)	0.0001 (6)
O9	0.0151 (7)	0.0199 (7)	0.0190 (8)	−0.0029 (6)	0.0019 (6)	0.0017 (6)
O10	0.0162 (7)	0.0186 (7)	0.0182 (7)	0.0005 (6)	0.0009 (6)	−0.0034 (6)
O11	0.0162 (7)	0.0239 (8)	0.0182 (8)	−0.0035 (6)	0.0053 (6)	−0.0019 (6)
O12	0.0181 (8)	0.0198 (7)	0.0245 (8)	−0.0009 (6)	−0.0011 (6)	0.0031 (6)
O13	0.0167 (8)	0.0200 (8)	0.0300 (9)	−0.0020 (6)	−0.0026 (7)	0.0071 (7)
O14	0.0313 (10)	0.0177 (8)	0.0215 (9)	−0.0013 (7)	0.0073 (7)	−0.0020 (7)
O15	0.0146 (8)	0.0355 (10)	0.0225 (9)	0.0035 (7)	0.0038 (7)	0.0086 (7)
O16	0.0368 (10)	0.0183 (8)	0.0255 (9)	0.0054 (7)	0.0086 (8)	0.0012 (7)
O17	0.0180 (8)	0.0274 (8)	0.0188 (8)	0.0024 (6)	0.0022 (6)	−0.0029 (7)
O18	0.0612 (15)	0.0243 (10)	0.0378 (12)	0.0150 (10)	−0.0127 (10)	−0.0055 (9)
C1	0.0190 (10)	0.0116 (8)	0.0166 (10)	−0.0019 (7)	0.0077 (8)	−0.0022 (7)
C2	0.0259 (11)	0.0146 (9)	0.0170 (10)	−0.0022 (8)	0.0014 (8)	−0.0007 (8)
C3	0.0386 (14)	0.0249 (12)	0.0167 (11)	−0.0049 (10)	0.0005 (10)	0.0013 (9)
C4	0.0427 (15)	0.0290 (12)	0.0172 (11)	−0.0104 (11)	0.0112 (10)	−0.0011 (9)
C5	0.0245 (12)	0.0223 (11)	0.0275 (12)	−0.0048 (9)	0.0125 (10)	−0.0064 (9)
C6	0.0210 (11)	0.0111 (9)	0.0226 (11)	−0.0008 (7)	0.0060 (8)	−0.0036 (8)
C7	0.0206 (11)	0.0222 (11)	0.0271 (12)	−0.0007 (9)	−0.0039 (9)	0.0043 (9)
C8A	0.0355 (18)	0.062 (2)	0.0366 (18)	−0.0048 (17)	−0.0148 (14)	0.0057 (17)
C9A	0.0241 (13)	0.0284 (14)	0.0421 (18)	0.0078 (11)	0.0002 (12)	−0.0011 (12)
C8B	0.0355 (18)	0.062 (2)	0.0366 (18)	−0.0048 (17)	−0.0148 (14)	0.0057 (17)
C9B	0.0241 (13)	0.0284 (14)	0.0421 (18)	0.0078 (11)	0.0002 (12)	−0.0011 (12)
C10	0.0153 (10)	0.0167 (10)	0.0331 (13)	−0.0005 (8)	0.0042 (9)	0.0001 (9)
C11	0.0208 (12)	0.0229 (12)	0.062 (2)	0.0043 (10)	0.0076 (12)	−0.0010 (12)
C12	0.0246 (13)	0.0245 (12)	0.0400 (15)	−0.0058 (10)	−0.0034 (11)	−0.0026 (11)
C13	0.0138 (9)	0.0113 (8)	0.0191 (10)	0.0002 (7)	0.0066 (8)	−0.0008 (7)
C14	0.0206 (11)	0.0187 (10)	0.0216 (11)	0.0004 (8)	0.0076 (9)	−0.0029 (8)
C15	0.0270 (12)	0.0211 (11)	0.0294 (13)	−0.0020 (9)	0.0076 (10)	−0.0089 (9)
C16	0.0284 (13)	0.0136 (10)	0.0382 (14)	0.0008 (9)	0.0123 (11)	−0.0049 (9)
C17	0.0278 (12)	0.0155 (10)	0.0313 (13)	0.0039 (9)	0.0090 (10)	0.0046 (9)
C18	0.0186 (10)	0.0171 (10)	0.0245 (11)	0.0019 (8)	0.0055 (9)	0.0007 (8)
C19	0.0294 (13)	0.0273 (12)	0.0197 (11)	0.0047 (10)	0.0048 (10)	−0.0007 (9)
C20	0.0398 (18)	0.061 (2)	0.0421 (18)	−0.0018 (16)	−0.0080 (14)	0.0086 (16)
C21	0.0400 (18)	0.082 (3)	0.051 (2)	0.0091 (17)	0.0118 (15)	0.0387 (19)
C22	0.0336 (14)	0.0235 (11)	0.0252 (12)	0.0064 (10)	−0.0028 (10)	0.0011 (9)
C23	0.0347 (16)	0.070 (2)	0.0283 (15)	−0.0040 (15)	0.0048 (12)	−0.0046 (14)
C24	0.0317 (16)	0.081 (3)	0.0366 (17)	−0.0148 (16)	0.0014 (13)	−0.0016 (17)
C25	0.0175 (10)	0.0150 (9)	0.0217 (11)	−0.0011 (8)	−0.0010 (8)	−0.0023 (8)
C26	0.0192 (11)	0.0198 (10)	0.0207 (11)	−0.0020 (8)	−0.0008 (8)	−0.0009 (8)
C27	0.0312 (12)	0.0191 (11)	0.0272 (12)	−0.0046 (9)	0.0002 (9)	0.0008 (9)
C28	0.0365 (14)	0.0174 (10)	0.0345 (14)	−0.0036 (10)	−0.0009 (11)	−0.0048 (10)
C29	0.0333 (13)	0.0238 (11)	0.0248 (12)	−0.0016 (10)	−0.0005 (10)	−0.0061 (9)
C30	0.0211 (11)	0.0222 (11)	0.0201 (11)	−0.0021 (8)	−0.0018 (9)	−0.0022 (8)
C31	0.0268 (12)	0.0222 (11)	0.0202 (11)	−0.0054 (9)	0.0009 (9)	−0.0003 (9)
C32	0.0379 (15)	0.0369 (15)	0.0289 (14)	0.0030 (12)	0.0073 (12)	−0.0032 (11)
C33	0.0474 (16)	0.0251 (12)	0.0216 (12)	−0.0058 (11)	0.0009 (11)	0.0013 (10)
C34	0.0362 (14)	0.0256 (12)	0.0194 (11)	−0.0023 (10)	0.0016 (10)	−0.0021 (9)
C35	0.0392 (16)	0.0490 (17)	0.0299 (15)	−0.0054 (13)	0.0083 (12)	−0.0018 (13)
C36	0.0455 (18)	0.0522 (18)	0.0254 (14)	0.0048 (14)	−0.0043 (13)	0.0075 (13)
C37	0.0267 (12)	0.0152 (10)	0.0302 (13)	0.0021 (9)	−0.0089 (10)	0.0026 (9)
C38	0.0380 (15)	0.0244 (12)	0.0240 (12)	−0.0003 (10)	−0.0063 (11)	0.0053 (10)
C39	0.065 (2)	0.0445 (18)	0.0308 (16)	−0.0084 (16)	−0.0151 (15)	0.0119 (13)
C40	0.074 (3)	0.049 (2)	0.052 (2)	−0.0071 (18)	−0.039 (2)	0.0150 (17)
C41	0.0404 (18)	0.0379 (16)	0.071 (2)	−0.0009 (14)	−0.0313 (17)	0.0119 (16)
C42	0.0280 (13)	0.0208 (12)	0.0532 (18)	0.0016 (10)	−0.0118 (12)	0.0041 (12)
C43	0.0336 (14)	0.0310 (13)	0.0263 (13)	−0.0006 (11)	0.0031 (11)	0.0075 (10)
C44	0.059 (2)	0.053 (2)	0.059 (2)	0.0018 (17)	0.0218 (19)	−0.0072 (18)
C45	0.0432 (17)	0.0364 (15)	0.0474 (18)	−0.0063 (13)	−0.0043 (14)	0.0105 (14)
C46	0.0205 (12)	0.0287 (13)	0.066 (2)	0.0033 (10)	−0.0020 (13)	−0.0003 (13)
C47A	0.0322 (18)	0.042 (2)	0.062 (3)	−0.0071 (15)	−0.0045 (17)	0.0011 (18)
C48A	0.047 (3)	0.043 (2)	0.078 (3)	0.015 (2)	−0.001 (2)	−0.003 (2)
C47B	0.0322 (18)	0.042 (2)	0.062 (3)	−0.0071 (15)	−0.0045 (17)	0.0011 (18)
C48B	0.047 (3)	0.043 (2)	0.078 (3)	0.015 (2)	−0.001 (2)	−0.003 (2)
C49	0.0127 (9)	0.0192 (10)	0.0200 (10)	0.0015 (7)	−0.0011 (8)	−0.0041 (8)
C50	0.0145 (10)	0.0218 (10)	0.0202 (11)	0.0043 (8)	−0.0001 (8)	0.0005 (8)
C51	0.0205 (11)	0.0263 (11)	0.0195 (11)	0.0064 (9)	0.0000 (9)	−0.0018 (9)
C52	0.0246 (12)	0.0224 (11)	0.0269 (12)	0.0086 (9)	−0.0031 (10)	−0.0057 (9)
C53	0.0254 (12)	0.0170 (10)	0.0297 (13)	0.0021 (9)	−0.0037 (10)	−0.0007 (9)
C54	0.0175 (10)	0.0200 (10)	0.0216 (11)	0.0003 (8)	−0.0017 (8)	0.0004 (8)
C55	0.0197 (11)	0.0232 (11)	0.0210 (11)	0.0020 (8)	0.0027 (9)	0.0021 (9)
C56	0.0287 (13)	0.0358 (14)	0.0300 (14)	0.0047 (11)	0.0096 (11)	0.0085 (11)
C57	0.0265 (13)	0.0261 (12)	0.0357 (14)	0.0059 (10)	0.0036 (11)	0.0077 (10)
C58	0.0300 (12)	0.0224 (11)	0.0259 (11)	−0.0065 (10)	0.0052 (9)	0.0022 (9)
C59	0.0318 (15)	0.0543 (19)	0.0479 (18)	−0.0062 (14)	0.0104 (13)	0.0009 (15)
C60	0.055 (2)	0.0515 (19)	0.0315 (16)	0.0076 (16)	0.0041 (14)	0.0156 (14)
C61	0.0187 (10)	0.0224 (10)	0.0163 (10)	−0.0023 (8)	0.0042 (8)	−0.0009 (8)
C62	0.0238 (12)	0.0322 (12)	0.0177 (11)	0.0007 (10)	0.0052 (9)	0.0026 (9)
C63	0.0327 (14)	0.0415 (15)	0.0236 (13)	−0.0003 (12)	0.0082 (11)	0.0095 (11)
C64	0.0244 (13)	0.0507 (17)	0.0322 (14)	−0.0052 (12)	0.0111 (11)	0.0090 (13)
C65	0.0173 (11)	0.0441 (15)	0.0300 (13)	0.0013 (10)	0.0041 (10)	0.0055 (11)
C66	0.0198 (11)	0.0271 (11)	0.0205 (11)	0.0013 (9)	0.0029 (9)	0.0015 (9)
C67	0.0251 (13)	0.0446 (15)	0.0210 (12)	0.0052 (11)	0.0033 (10)	0.0089 (11)
C68	0.064 (2)	0.066 (2)	0.050 (2)	0.006 (2)	−0.0306 (19)	−0.0015 (18)
C69	0.0466 (19)	0.065 (2)	0.0427 (18)	0.0116 (17)	−0.0008 (15)	0.0278 (17)
C70	0.0227 (12)	0.0299 (12)	0.0247 (12)	0.0027 (9)	0.0017 (9)	0.0066 (10)
C71	0.0435 (17)	0.0537 (19)	0.0251 (14)	−0.0082 (14)	−0.0031 (12)	0.0063 (13)
C72	0.082 (3)	0.0376 (17)	0.0381 (18)	0.0191 (17)	0.0035 (17)	0.0054 (14)
C73	0.0319 (13)	0.0217 (11)	0.0276 (13)	−0.0001 (10)	−0.0065 (10)	0.0041 (9)
C74	0.0300 (13)	0.0193 (11)	0.0336 (14)	0.0018 (9)	0.0017 (11)	−0.0076 (10)
C75	0.0218 (12)	0.0299 (13)	0.0377 (15)	0.0060 (10)	−0.0025 (11)	0.0085 (11)
C76	0.0421 (16)	0.0195 (11)	0.0345 (14)	−0.0027 (10)	0.0041 (12)	0.0058 (10)
C77	0.0224 (12)	0.0364 (14)	0.0293 (13)	0.0051 (10)	0.0053 (10)	−0.0097 (11)
C78	0.061 (2)	0.0220 (13)	0.058 (2)	0.0061 (13)	−0.0013 (17)	−0.0063 (13)

### Geometric parameters (Å, º)

**Table:**

Eu1—O1	2.3915 (14)	C33—H33C	0.9800
Eu1—O5	2.3166 (15)	C34—C35	1.528 (4)
Eu1—O9	2.3525 (15)	C34—C36	1.533 (4)
Eu1—O13	2.4374 (16)	C34—H34A	1.0000
Eu1—O14	2.4933 (16)	C35—H35A	0.9800
Eu1—O15	2.4312 (17)	C35—H35B	0.9800
Eu1—O16	2.4664 (17)	C35—H35C	0.9800
Eu1—O17	2.4665 (16)	C36—H36A	0.9800
P1—O1	1.4963 (16)	C36—H36B	0.9800
P1—O2	1.5991 (15)	C36—H36C	0.9800
P1—O3	1.5935 (16)	C37—C38	1.394 (4)
P1—O4	1.4922 (16)	C37—C42	1.402 (4)
P2—O5	1.4972 (16)	C38—C39	1.401 (4)
P2—O6	1.5938 (16)	C38—C43	1.517 (4)
P2—O7	1.5978 (16)	C39—C40	1.381 (5)
P2—O8	1.4923 (17)	C39—H39A	0.9500
P3—O9	1.5010 (16)	C40—C41	1.372 (5)
P3—O10	1.6007 (16)	C40—H40A	0.9500
P3—O11	1.5970 (16)	C41—C42	1.391 (4)
P3—O12	1.4855 (17)	C41—H41A	0.9500
O2—C1	1.413 (2)	C42—C46	1.517 (4)
O3—C13	1.413 (2)	C43—C44	1.522 (4)
O6—C25	1.410 (3)	C43—C45	1.531 (4)
O7—C37	1.402 (3)	C43—H43A	1.0000
O10—C49	1.411 (3)	C44—H44A	0.9800
O11—C61	1.406 (3)	C44—H44B	0.9800
O13—C73	1.420 (3)	C44—H44C	0.9800
O13—H79	0.81 (3)	C45—H45A	0.9800
O14—C74	1.434 (3)	C45—H45B	0.9800
O14—H80	0.76 (3)	C45—H45C	0.9800
O15—C75	1.414 (3)	C46—C48B	1.527 (4)
O15—H81	0.82 (3)	C46—C47A	1.527 (3)
O16—C76	1.430 (3)	C46—C48A	1.527 (3)
O16—H82	0.82 (3)	C46—C47B	1.530 (4)
O17—C77	1.440 (3)	C46—H46A	1.0000
O17—H83	0.82 (3)	C46—H46B	1.0000
O18—C78	1.383 (3)	C47A—H47A	0.9800
O18—H84	0.82 (4)	C47A—H47B	0.9800
C1—C2	1.395 (3)	C47A—H47C	0.9800
C1—C6	1.401 (3)	C48A—H48A	0.9800
C2—C3	1.398 (3)	C48A—H48B	0.9800
C2—C7	1.518 (3)	C48A—H48C	0.9800
C3—C4	1.381 (4)	C47B—H47D	0.9800
C3—H3A	0.9500	C47B—H47E	0.9800
C4—C5	1.377 (4)	C47B—H47F	0.9800
C4—H4A	0.9500	C48B—H48D	0.9800
C5—C6	1.395 (3)	C48B—H48E	0.9800
C5—H5A	0.9500	C48B—H48F	0.9800
C6—C10	1.513 (3)	C49—C54	1.394 (3)
C7—C8A	1.531 (3)	C49—C50	1.399 (3)
C7—C8B	1.532 (4)	C50—C51	1.390 (3)
C7—C9B	1.532 (4)	C50—C55	1.521 (3)
C7—C9A	1.533 (3)	C51—C52	1.388 (3)
C7—H7A	1.0000	C51—H51A	0.9500
C7—H7B	1.0000	C52—C53	1.381 (4)
C8A—H8A	0.9800	C52—H52A	0.9500
C8A—H8B	0.9800	C53—C54	1.396 (3)
C8A—H8C	0.9800	C53—H53A	0.9500
C9A—H9A	0.9800	C54—C58	1.516 (3)
C9A—H9B	0.9800	C55—C56	1.527 (3)
C9A—H9C	0.9800	C55—C57	1.534 (3)
C8B—H8D	0.9800	C55—H55A	1.0000
C8B—H8E	0.9800	C56—H56A	0.9800
C8B—H8F	0.9800	C56—H56B	0.9800
C9B—H9D	0.9800	C56—H56C	0.9800
C9B—H9E	0.9800	C57—H57A	0.9800
C9B—H9F	0.9800	C57—H57B	0.9800
C10—C11	1.535 (3)	C57—H57C	0.9800
C10—C12	1.537 (3)	C58—C60	1.524 (4)
C10—H10A	1.0000	C58—C59	1.525 (4)
C11—H11A	0.9800	C58—H58A	1.0000
C11—H11B	0.9800	C59—H59A	0.9800
C11—H11C	0.9800	C59—H59B	0.9800
C12—H12A	0.9800	C59—H59C	0.9800
C12—H12B	0.9800	C60—H60A	0.9800
C12—H12C	0.9800	C60—H60B	0.9800
C13—C14	1.392 (3)	C60—H60C	0.9800
C13—C18	1.396 (3)	C61—C66	1.394 (3)
C14—C15	1.402 (3)	C61—C62	1.401 (3)
C14—C19	1.524 (3)	C62—C63	1.387 (3)
C15—C16	1.376 (4)	C62—C67	1.513 (4)
C15—H15A	0.9500	C63—C64	1.383 (4)
C16—C17	1.383 (4)	C63—H63A	0.9500
C16—H16A	0.9500	C64—C65	1.380 (4)
C17—C18	1.401 (3)	C64—H64A	0.9500
C17—H17A	0.9500	C65—C66	1.396 (3)
C18—C22	1.517 (3)	C65—H65A	0.9500
C19—C20	1.517 (4)	C66—C70	1.516 (3)
C19—C21	1.527 (4)	C67—C68	1.522 (5)
C19—H19A	1.0000	C67—C69	1.528 (4)
C20—H20A	0.9800	C67—H67A	1.0000
C20—H20B	0.9800	C68—H68A	0.9800
C20—H20C	0.9800	C68—H68B	0.9800
C21—H21A	0.9800	C68—H68C	0.9800
C21—H21B	0.9800	C69—H69A	0.9800
C21—H21C	0.9800	C69—H69B	0.9800
C22—C23	1.498 (4)	C69—H69C	0.9800
C22—C24	1.533 (4)	C70—C71	1.521 (4)
C22—H22A	1.0000	C70—C72	1.529 (4)
C23—H23A	0.9800	C70—H70A	1.0000
C23—H23B	0.9800	C71—H71A	0.9800
C23—H23C	0.9800	C71—H71B	0.9800
C24—H24A	0.9800	C71—H71C	0.9800
C24—H24B	0.9800	C72—H72A	0.9800
C24—H24C	0.9800	C72—H72B	0.9800
C25—C26	1.397 (3)	C72—H72C	0.9800
C25—C30	1.397 (3)	C73—H73A	0.9800
C26—C27	1.401 (3)	C73—H73B	0.9800
C26—C31	1.521 (3)	C73—H73C	0.9800
C27—C28	1.388 (4)	C74—H74A	0.9800
C27—H27A	0.9500	C74—H74B	0.9800
C28—C29	1.376 (4)	C74—H74C	0.9800
C28—H28A	0.9500	C75—H75A	0.9800
C29—C30	1.400 (3)	C75—H75B	0.9800
C29—H29A	0.9500	C75—H75C	0.9800
C30—C34	1.515 (3)	C76—H76A	0.9800
C31—C32	1.532 (4)	C76—H76B	0.9800
C31—C33	1.532 (3)	C76—H76C	0.9800
C31—H31A	1.0000	C77—H77A	0.9800
C32—H32A	0.9800	C77—H77B	0.9800
C32—H32B	0.9800	C77—H77C	0.9800
C32—H32C	0.9800	C78—H78A	0.9800
C33—H33A	0.9800	C78—H78B	0.9800
C33—H33B	0.9800	C78—H78C	0.9800
			
O5—Eu1—O9	138.83 (5)	C31—C33—H33C	109.5
O5—Eu1—O1	79.87 (5)	H33A—C33—H33C	109.5
O9—Eu1—O1	140.28 (5)	H33B—C33—H33C	109.5
O5—Eu1—O15	146.42 (6)	C30—C34—C35	111.5 (2)
O9—Eu1—O15	71.23 (6)	C30—C34—C36	110.4 (2)
O1—Eu1—O15	75.69 (5)	C35—C34—C36	111.0 (2)
O5—Eu1—O13	77.37 (6)	C30—C34—H34A	107.9
O9—Eu1—O13	73.50 (5)	C35—C34—H34A	107.9
O1—Eu1—O13	119.54 (5)	C36—C34—H34A	107.9
O15—Eu1—O13	135.11 (6)	C34—C35—H35A	109.5
O5—Eu1—O16	82.25 (6)	C34—C35—H35B	109.5
O9—Eu1—O16	108.35 (6)	H35A—C35—H35B	109.5
O1—Eu1—O16	80.44 (6)	C34—C35—H35C	109.5
O15—Eu1—O16	71.35 (6)	H35A—C35—H35C	109.5
O13—Eu1—O16	147.71 (6)	H35B—C35—H35C	109.5
O5—Eu1—O17	74.43 (6)	C34—C36—H36A	109.5
O9—Eu1—O17	72.91 (6)	C34—C36—H36B	109.5
O1—Eu1—O17	142.29 (6)	H36A—C36—H36B	109.5
O15—Eu1—O17	113.10 (6)	C34—C36—H36C	109.5
O13—Eu1—O17	81.23 (6)	H36A—C36—H36C	109.5
O16—Eu1—O17	69.24 (6)	H36B—C36—H36C	109.5
O5—Eu1—O14	119.42 (6)	C38—C37—C42	123.6 (2)
O9—Eu1—O14	78.32 (6)	C38—C37—O7	118.8 (2)
O1—Eu1—O14	71.84 (5)	C42—C37—O7	117.3 (2)
O15—Eu1—O14	73.96 (6)	C37—C38—C39	116.7 (3)
O13—Eu1—O14	72.45 (6)	C37—C38—C43	123.4 (2)
O16—Eu1—O14	139.83 (6)	C39—C38—C43	119.9 (3)
O17—Eu1—O14	145.44 (6)	C40—C39—C38	121.2 (3)
O5—Eu1—P1	97.10 (4)	C40—C39—H39A	119.4
O9—Eu1—P1	122.99 (4)	C38—C39—H39A	119.4
O1—Eu1—P1	17.33 (4)	C41—C40—C39	120.1 (3)
O15—Eu1—P1	60.84 (4)	C41—C40—H40A	120.0
O13—Eu1—P1	122.42 (4)	C39—C40—H40A	120.0
O16—Eu1—P1	84.56 (4)	C40—C41—C42	122.1 (3)
O17—Eu1—P1	153.18 (4)	C40—C41—H41A	119.0
O14—Eu1—P1	60.87 (4)	C42—C41—H41A	119.0
O4—P1—O1	114.89 (9)	C41—C42—C37	116.3 (3)
O4—P1—O3	109.70 (9)	C41—C42—C46	121.1 (3)
O1—P1—O3	112.59 (9)	C37—C42—C46	122.5 (3)
O4—P1—O2	113.71 (9)	C38—C43—C44	112.0 (3)
O1—P1—O2	105.79 (8)	C38—C43—C45	110.2 (2)
O3—P1—O2	99.08 (8)	C44—C43—C45	111.2 (3)
O8—P2—O5	116.23 (9)	C38—C43—H43A	107.8
O8—P2—O6	111.29 (9)	C44—C43—H43A	107.8
O5—P2—O6	110.43 (9)	C45—C43—H43A	107.8
O8—P2—O7	113.19 (9)	C43—C44—H44A	109.5
O5—P2—O7	103.61 (9)	C43—C44—H44B	109.5
O6—P2—O7	100.80 (9)	H44A—C44—H44B	109.5
O12—P3—O9	116.11 (9)	C43—C44—H44C	109.5
O12—P3—O11	113.92 (9)	H44A—C44—H44C	109.5
O9—P3—O11	103.52 (9)	H44B—C44—H44C	109.5
O12—P3—O10	110.71 (9)	C43—C45—H45A	109.5
O9—P3—O10	110.16 (9)	C43—C45—H45B	109.5
O11—P3—O10	101.24 (8)	H45A—C45—H45B	109.5
P1—O1—Eu1	134.23 (9)	C43—C45—H45C	109.5
C1—O2—P1	123.95 (13)	H45A—C45—H45C	109.5
C13—O3—P1	124.19 (13)	H45B—C45—H45C	109.5
P2—O5—Eu1	146.84 (10)	C42—C46—C48B	102.4 (15)
C25—O6—P2	128.96 (14)	C42—C46—C47A	112.7 (3)
C37—O7—P2	127.11 (14)	C42—C46—C48A	112.6 (3)
P3—O9—Eu1	142.27 (9)	C47A—C46—C48A	110.1 (3)
C49—O10—P3	124.05 (14)	C42—C46—C47B	100.1 (11)
C61—O11—P3	128.60 (14)	C48B—C46—C47B	108.9 (17)
C73—O13—Eu1	142.50 (15)	C42—C46—H46A	107.0
C73—O13—H79	104 (2)	C47A—C46—H46A	107.0
Eu1—O13—H79	112 (2)	C48A—C46—H46A	107.0
C74—O14—Eu1	136.77 (15)	C42—C46—H46B	114.6
C74—O14—H80	112 (2)	C48B—C46—H46B	114.6
Eu1—O14—H80	111 (2)	C47B—C46—H46B	114.6
C75—O15—Eu1	133.14 (15)	C46—C47A—H47A	109.5
C75—O15—H81	114 (2)	C46—C47A—H47B	109.5
Eu1—O15—H81	113 (2)	H47A—C47A—H47B	109.5
C76—O16—Eu1	132.06 (15)	C46—C47A—H47C	109.5
C76—O16—H82	108 (2)	H47A—C47A—H47C	109.5
Eu1—O16—H82	116 (2)	H47B—C47A—H47C	109.5
C77—O17—Eu1	123.54 (14)	C46—C48A—H48A	109.5
C77—O17—H83	112 (2)	C46—C48A—H48B	109.5
Eu1—O17—H83	114 (2)	H48A—C48A—H48B	109.5
C78—O18—H84	112 (3)	C46—C48A—H48C	109.5
C2—C1—C6	123.4 (2)	H48A—C48A—H48C	109.5
C2—C1—O2	118.23 (19)	H48B—C48A—H48C	109.5
C6—C1—O2	118.16 (19)	C46—C47B—H47D	109.5
C1—C2—C3	117.0 (2)	C46—C47B—H47E	109.5
C1—C2—C7	120.36 (19)	H47D—C47B—H47E	109.5
C3—C2—C7	122.4 (2)	C46—C47B—H47F	109.5
C4—C3—C2	121.1 (2)	H47D—C47B—H47F	109.5
C4—C3—H3A	119.5	H47E—C47B—H47F	109.5
C2—C3—H3A	119.5	C46—C48B—H48D	109.5
C5—C4—C3	120.2 (2)	C46—C48B—H48E	109.5
C5—C4—H4A	119.9	H48D—C48B—H48E	109.5
C3—C4—H4A	119.9	C46—C48B—H48F	109.5
C4—C5—C6	121.6 (2)	H48D—C48B—H48F	109.5
C4—C5—H5A	119.2	H48E—C48B—H48F	109.5
C6—C5—H5A	119.2	C54—C49—C50	122.8 (2)
C5—C6—C1	116.6 (2)	C54—C49—O10	119.42 (19)
C5—C6—C10	119.8 (2)	C50—C49—O10	117.56 (19)
C1—C6—C10	123.5 (2)	C51—C50—C49	117.7 (2)
C2—C7—C8A	114.2 (2)	C51—C50—C55	121.6 (2)
C2—C7—C8B	107.8 (17)	C49—C50—C55	120.6 (2)
C2—C7—C9B	104.7 (13)	C52—C51—C50	120.9 (2)
C8B—C7—C9B	113 (2)	C52—C51—H51A	119.5
C2—C7—C9A	108.89 (19)	C50—C51—H51A	119.5
C8A—C7—C9A	110.2 (2)	C53—C52—C51	120.0 (2)
C2—C7—H7A	107.8	C53—C52—H52A	120.0
C8A—C7—H7A	107.8	C51—C52—H52A	120.0
C9A—C7—H7A	107.8	C52—C53—C54	121.4 (2)
C2—C7—H7B	110.3	C52—C53—H53A	119.3
C8B—C7—H7B	110.3	C54—C53—H53A	119.3
C9B—C7—H7B	110.3	C49—C54—C53	117.2 (2)
C7—C8A—H8A	109.5	C49—C54—C58	122.9 (2)
C7—C8A—H8B	109.5	C53—C54—C58	119.9 (2)
H8A—C8A—H8B	109.5	C50—C55—C56	114.4 (2)
C7—C8A—H8C	109.5	C50—C55—C57	108.37 (19)
H8A—C8A—H8C	109.5	C56—C55—C57	110.0 (2)
H8B—C8A—H8C	109.5	C50—C55—H55A	108.0
C7—C9A—H9A	109.5	C56—C55—H55A	108.0
C7—C9A—H9B	109.5	C57—C55—H55A	108.0
H9A—C9A—H9B	109.5	C55—C56—H56A	109.5
C7—C9A—H9C	109.5	C55—C56—H56B	109.5
H9A—C9A—H9C	109.5	H56A—C56—H56B	109.5
H9B—C9A—H9C	109.5	C55—C56—H56C	109.5
C7—C8B—H8D	109.5	H56A—C56—H56C	109.5
C7—C8B—H8E	109.5	H56B—C56—H56C	109.5
H8D—C8B—H8E	109.5	C55—C57—H57A	109.5
C7—C8B—H8F	109.5	C55—C57—H57B	109.5
H8D—C8B—H8F	109.5	H57A—C57—H57B	109.5
H8E—C8B—H8F	109.5	C55—C57—H57C	109.5
C7—C9B—H9D	109.5	H57A—C57—H57C	109.5
C7—C9B—H9E	109.5	H57B—C57—H57C	109.5
H9D—C9B—H9E	109.5	C54—C58—C60	111.4 (2)
C7—C9B—H9F	109.5	C54—C58—C59	111.3 (2)
H9D—C9B—H9F	109.5	C60—C58—C59	111.0 (2)
H9E—C9B—H9F	109.5	C54—C58—H58A	107.7
C6—C10—C11	111.7 (2)	C60—C58—H58A	107.7
C6—C10—C12	110.65 (19)	C59—C58—H58A	107.7
C11—C10—C12	110.1 (2)	C58—C59—H59A	109.5
C6—C10—H10A	108.1	C58—C59—H59B	109.5
C11—C10—H10A	108.1	H59A—C59—H59B	109.5
C12—C10—H10A	108.1	C58—C59—H59C	109.5
C10—C11—H11A	109.5	H59A—C59—H59C	109.5
C10—C11—H11B	109.5	H59B—C59—H59C	109.5
H11A—C11—H11B	109.5	C58—C60—H60A	109.5
C10—C11—H11C	109.5	C58—C60—H60B	109.5
H11A—C11—H11C	109.5	H60A—C60—H60B	109.5
H11B—C11—H11C	109.5	C58—C60—H60C	109.5
C10—C12—H12A	109.5	H60A—C60—H60C	109.5
C10—C12—H12B	109.5	H60B—C60—H60C	109.5
H12A—C12—H12B	109.5	C66—C61—C62	123.6 (2)
C10—C12—H12C	109.5	C66—C61—O11	117.44 (19)
H12A—C12—H12C	109.5	C62—C61—O11	118.8 (2)
H12B—C12—H12C	109.5	C63—C62—C61	116.4 (2)
C14—C13—C18	123.79 (19)	C63—C62—C67	122.2 (2)
C14—C13—O3	119.33 (19)	C61—C62—C67	121.4 (2)
C18—C13—O3	116.71 (19)	C64—C63—C62	122.1 (2)
C13—C14—C15	116.3 (2)	C64—C63—H63A	119.0
C13—C14—C19	122.8 (2)	C62—C63—H63A	119.0
C15—C14—C19	120.8 (2)	C65—C64—C63	119.7 (2)
C16—C15—C14	122.0 (2)	C65—C64—H64A	120.1
C16—C15—H15A	119.0	C63—C64—H64A	120.1
C14—C15—H15A	119.0	C64—C65—C66	121.3 (2)
C15—C16—C17	119.8 (2)	C64—C65—H65A	119.4
C15—C16—H16A	120.1	C66—C65—H65A	119.4
C17—C16—H16A	120.1	C61—C66—C65	117.0 (2)
C16—C17—C18	121.2 (2)	C61—C66—C70	122.8 (2)
C16—C17—H17A	119.4	C65—C66—C70	120.2 (2)
C18—C17—H17A	119.4	C62—C67—C68	111.0 (2)
C13—C18—C17	116.9 (2)	C62—C67—C69	113.9 (2)
C13—C18—C22	122.3 (2)	C68—C67—C69	110.4 (3)
C17—C18—C22	120.8 (2)	C62—C67—H67A	107.0
C20—C19—C14	112.7 (2)	C68—C67—H67A	107.0
C20—C19—C21	110.2 (3)	C69—C67—H67A	107.0
C14—C19—C21	110.7 (2)	C67—C68—H68A	109.5
C20—C19—H19A	107.7	C67—C68—H68B	109.5
C14—C19—H19A	107.7	H68A—C68—H68B	109.5
C21—C19—H19A	107.7	C67—C68—H68C	109.5
C19—C20—H20A	109.5	H68A—C68—H68C	109.5
C19—C20—H20B	109.5	H68B—C68—H68C	109.5
H20A—C20—H20B	109.5	C67—C69—H69A	109.5
C19—C20—H20C	109.5	C67—C69—H69B	109.5
H20A—C20—H20C	109.5	H69A—C69—H69B	109.5
H20B—C20—H20C	109.5	C67—C69—H69C	109.5
C19—C21—H21A	109.5	H69A—C69—H69C	109.5
C19—C21—H21B	109.5	H69B—C69—H69C	109.5
H21A—C21—H21B	109.5	C66—C70—C71	111.8 (2)
C19—C21—H21C	109.5	C66—C70—C72	111.1 (2)
H21A—C21—H21C	109.5	C71—C70—C72	111.1 (2)
H21B—C21—H21C	109.5	C66—C70—H70A	107.5
C23—C22—C18	112.3 (2)	C71—C70—H70A	107.5
C23—C22—C24	110.5 (2)	C72—C70—H70A	107.5
C18—C22—C24	111.1 (2)	C70—C71—H71A	109.5
C23—C22—H22A	107.6	C70—C71—H71B	109.5
C18—C22—H22A	107.6	H71A—C71—H71B	109.5
C24—C22—H22A	107.6	C70—C71—H71C	109.5
C22—C23—H23A	109.5	H71A—C71—H71C	109.5
C22—C23—H23B	109.5	H71B—C71—H71C	109.5
H23A—C23—H23B	109.5	C70—C72—H72A	109.5
C22—C23—H23C	109.5	C70—C72—H72B	109.5
H23A—C23—H23C	109.5	H72A—C72—H72B	109.5
H23B—C23—H23C	109.5	C70—C72—H72C	109.5
C22—C24—H24A	109.5	H72A—C72—H72C	109.5
C22—C24—H24B	109.5	H72B—C72—H72C	109.5
H24A—C24—H24B	109.5	O13—C73—H73A	109.5
C22—C24—H24C	109.5	O13—C73—H73B	109.5
H24A—C24—H24C	109.5	H73A—C73—H73B	109.5
H24B—C24—H24C	109.5	O13—C73—H73C	109.5
C26—C25—C30	123.4 (2)	H73A—C73—H73C	109.5
C26—C25—O6	117.54 (19)	H73B—C73—H73C	109.5
C30—C25—O6	118.8 (2)	O14—C74—H74A	109.5
C25—C26—C27	116.9 (2)	O14—C74—H74B	109.5
C25—C26—C31	121.1 (2)	H74A—C74—H74B	109.5
C27—C26—C31	122.0 (2)	O14—C74—H74C	109.5
C28—C27—C26	121.3 (2)	H74A—C74—H74C	109.5
C28—C27—H27A	119.3	H74B—C74—H74C	109.5
C26—C27—H27A	119.3	O15—C75—H75A	109.5
C29—C28—C27	119.9 (2)	O15—C75—H75B	109.5
C29—C28—H28A	120.1	H75A—C75—H75B	109.5
C27—C28—H28A	120.1	O15—C75—H75C	109.5
C28—C29—C30	121.6 (2)	H75A—C75—H75C	109.5
C28—C29—H29A	119.2	H75B—C75—H75C	109.5
C30—C29—H29A	119.2	O16—C76—H76A	109.5
C25—C30—C29	116.9 (2)	O16—C76—H76B	109.5
C25—C30—C34	123.1 (2)	H76A—C76—H76B	109.5
C29—C30—C34	119.9 (2)	O16—C76—H76C	109.5
C26—C31—C32	111.5 (2)	H76A—C76—H76C	109.5
C26—C31—C33	112.9 (2)	H76B—C76—H76C	109.5
C32—C31—C33	109.5 (2)	O17—C77—H77A	109.5
C26—C31—H31A	107.6	O17—C77—H77B	109.5
C32—C31—H31A	107.6	H77A—C77—H77B	109.5
C33—C31—H31A	107.6	O17—C77—H77C	109.5
C31—C32—H32A	109.5	H77A—C77—H77C	109.5
C31—C32—H32B	109.5	H77B—C77—H77C	109.5
H32A—C32—H32B	109.5	O18—C78—H78A	109.5
C31—C32—H32C	109.5	O18—C78—H78B	109.5
H32A—C32—H32C	109.5	H78A—C78—H78B	109.5
H32B—C32—H32C	109.5	O18—C78—H78C	109.5
C31—C33—H33A	109.5	H78A—C78—H78C	109.5
C31—C33—H33B	109.5	H78B—C78—H78C	109.5
H33A—C33—H33B	109.5		
			
O4—P1—O1—Eu1	2.86 (16)	C26—C25—C30—C29	0.2 (4)
O3—P1—O1—Eu1	−123.68 (11)	O6—C25—C30—C29	173.7 (2)
O2—P1—O1—Eu1	129.15 (11)	C26—C25—C30—C34	−177.5 (2)
O4—P1—O2—C1	−67.23 (18)	O6—C25—C30—C34	−4.0 (3)
O1—P1—O2—C1	165.77 (16)	C28—C29—C30—C25	−0.9 (4)
O3—P1—O2—C1	49.06 (17)	C28—C29—C30—C34	176.9 (2)
Eu1—P1—O2—C1	−168.45 (13)	C25—C26—C31—C32	−71.5 (3)
O4—P1—O3—C13	−46.38 (18)	C27—C26—C31—C32	107.5 (3)
O1—P1—O3—C13	82.90 (18)	C25—C26—C31—C33	164.7 (2)
O2—P1—O3—C13	−165.70 (16)	C27—C26—C31—C33	−16.3 (3)
Eu1—P1—O3—C13	53.75 (18)	C25—C30—C34—C35	−126.2 (3)
O8—P2—O5—Eu1	−19.6 (2)	C29—C30—C34—C35	56.2 (3)
O6—P2—O5—Eu1	−147.60 (15)	C25—C30—C34—C36	110.0 (3)
O7—P2—O5—Eu1	105.20 (17)	C29—C30—C34—C36	−67.7 (3)
O8—P2—O6—C25	−32.1 (2)	P2—O7—C37—C38	92.1 (2)
O5—P2—O6—C25	98.58 (19)	P2—O7—C37—C42	−94.4 (2)
O7—P2—O6—C25	−152.36 (18)	C42—C37—C38—C39	4.4 (4)
O8—P2—O7—C37	−66.8 (2)	O7—C37—C38—C39	177.5 (2)
O5—P2—O7—C37	166.42 (19)	C42—C37—C38—C43	−172.3 (2)
O6—P2—O7—C37	52.1 (2)	O7—C37—C38—C43	0.8 (4)
O12—P3—O9—Eu1	−26.26 (19)	C37—C38—C39—C40	−1.7 (5)
O11—P3—O9—Eu1	99.35 (15)	C43—C38—C39—C40	175.1 (3)
O10—P3—O9—Eu1	−153.09 (13)	C38—C39—C40—C41	−1.0 (5)
O12—P3—O10—C49	−25.88 (19)	C39—C40—C41—C42	1.3 (5)
O9—P3—O10—C49	103.91 (17)	C40—C41—C42—C37	1.1 (5)
O11—P3—O10—C49	−147.02 (16)	C40—C41—C42—C46	−176.4 (3)
O12—P3—O11—C61	−71.7 (2)	C38—C37—C42—C41	−4.1 (4)
O9—P3—O11—C61	161.31 (18)	O7—C37—C42—C41	−177.3 (2)
O10—P3—O11—C61	47.2 (2)	C38—C37—C42—C46	173.4 (2)
P1—O2—C1—C2	92.3 (2)	O7—C37—C42—C46	0.2 (3)
P1—O2—C1—C6	−93.2 (2)	C37—C38—C43—C44	−121.9 (3)
C6—C1—C2—C3	3.8 (3)	C39—C38—C43—C44	61.5 (4)
O2—C1—C2—C3	178.07 (18)	C37—C38—C43—C45	113.8 (3)
C6—C1—C2—C7	−170.50 (19)	C39—C38—C43—C45	−62.8 (3)
O2—C1—C2—C7	3.8 (3)	C41—C42—C46—C48B	93.3 (14)
C1—C2—C3—C4	−1.5 (3)	C37—C42—C46—C48B	−84.1 (14)
C7—C2—C3—C4	172.7 (2)	C41—C42—C46—C47A	−60.2 (4)
C2—C3—C4—C5	−0.8 (4)	C37—C42—C46—C47A	122.4 (3)
C3—C4—C5—C6	0.8 (4)	C41—C42—C46—C48A	65.1 (4)
C4—C5—C6—C1	1.3 (3)	C37—C42—C46—C48A	−112.3 (3)
C4—C5—C6—C10	−176.4 (2)	C41—C42—C46—C47B	−18.9 (11)
C2—C1—C6—C5	−3.7 (3)	C37—C42—C46—C47B	163.8 (11)
O2—C1—C6—C5	−178.02 (18)	P3—O10—C49—C54	87.5 (2)
C2—C1—C6—C10	173.9 (2)	P3—O10—C49—C50	−97.3 (2)
O2—C1—C6—C10	−0.4 (3)	C54—C49—C50—C51	−0.7 (3)
C1—C2—C7—C8A	−159.8 (2)	O10—C49—C50—C51	−175.72 (19)
C3—C2—C7—C8A	26.2 (3)	C54—C49—C50—C55	174.3 (2)
C1—C2—C7—C8B	−123.2 (19)	O10—C49—C50—C55	−0.7 (3)
C3—C2—C7—C8B	62.8 (19)	C49—C50—C51—C52	0.5 (3)
C1—C2—C7—C9B	115.7 (15)	C55—C50—C51—C52	−174.5 (2)
C3—C2—C7—C9B	−58.3 (15)	C50—C51—C52—C53	0.0 (4)
C1—C2—C7—C9A	76.6 (3)	C51—C52—C53—C54	−0.2 (4)
C3—C2—C7—C9A	−97.4 (3)	C50—C49—C54—C53	0.5 (3)
C5—C6—C10—C11	−60.3 (3)	O10—C49—C54—C53	175.44 (19)
C1—C6—C10—C11	122.1 (2)	C50—C49—C54—C58	−177.6 (2)
C5—C6—C10—C12	62.6 (3)	O10—C49—C54—C58	−2.7 (3)
C1—C6—C10—C12	−114.9 (2)	C52—C53—C54—C49	−0.1 (3)
P1—O3—C13—C14	83.9 (2)	C52—C53—C54—C58	178.1 (2)
P1—O3—C13—C18	−100.6 (2)	C51—C50—C55—C56	−26.9 (3)
C18—C13—C14—C15	3.2 (3)	C49—C50—C55—C56	158.3 (2)
O3—C13—C14—C15	178.32 (19)	C51—C50—C55—C57	96.2 (3)
C18—C13—C14—C19	−173.7 (2)	C49—C50—C55—C57	−78.6 (3)
O3—C13—C14—C19	1.4 (3)	C49—C54—C58—C60	−124.1 (3)
C13—C14—C15—C16	−1.0 (3)	C53—C54—C58—C60	57.8 (3)
C19—C14—C15—C16	175.9 (2)	C49—C54—C58—C59	111.5 (3)
C14—C15—C16—C17	−0.9 (4)	C53—C54—C58—C59	−66.6 (3)
C15—C16—C17—C18	0.7 (4)	P3—O11—C61—C66	−96.0 (2)
C14—C13—C18—C17	−3.4 (3)	P3—O11—C61—C62	89.1 (2)
O3—C13—C18—C17	−178.59 (19)	C66—C61—C62—C63	2.7 (4)
C14—C13—C18—C22	175.2 (2)	O11—C61—C62—C63	177.3 (2)
O3—C13—C18—C22	−0.1 (3)	C66—C61—C62—C67	−174.9 (2)
C16—C17—C18—C13	1.3 (3)	O11—C61—C62—C67	−0.4 (3)
C16—C17—C18—C22	−177.3 (2)	C61—C62—C63—C64	−1.8 (4)
C13—C14—C19—C20	−130.5 (3)	C67—C62—C63—C64	175.8 (3)
C15—C14—C19—C20	52.8 (3)	C62—C63—C64—C65	0.1 (5)
C13—C14—C19—C21	105.6 (3)	C63—C64—C65—C66	0.8 (5)
C15—C14—C19—C21	−71.1 (3)	C62—C61—C66—C65	−1.8 (4)
C13—C18—C22—C23	118.8 (3)	O11—C61—C66—C65	−176.5 (2)
C17—C18—C22—C23	−62.7 (3)	C62—C61—C66—C70	176.5 (2)
C13—C18—C22—C24	−116.8 (3)	O11—C61—C66—C70	1.9 (3)
C17—C18—C22—C24	61.7 (3)	C64—C65—C66—C61	0.0 (4)
P2—O6—C25—C26	−98.9 (2)	C64—C65—C66—C70	−178.4 (3)
P2—O6—C25—C30	87.2 (2)	C63—C62—C67—C68	−105.5 (3)
C30—C25—C26—C27	0.4 (3)	C61—C62—C67—C68	72.0 (3)
O6—C25—C26—C27	−173.2 (2)	C63—C62—C67—C69	19.9 (4)
C30—C25—C26—C31	179.5 (2)	C61—C62—C67—C69	−162.6 (3)
O6—C25—C26—C31	5.9 (3)	C61—C66—C70—C71	127.1 (3)
C25—C26—C27—C28	−0.4 (4)	C65—C66—C70—C71	−54.5 (3)
C31—C26—C27—C28	−179.5 (2)	C61—C66—C70—C72	−108.0 (3)
C26—C27—C28—C29	−0.2 (4)	C65—C66—C70—C72	70.3 (3)
C27—C28—C29—C30	0.9 (4)		

### Hydrogen-bond geometry (Å, º)

**Table:**

*D*—H···*A*	*D*—H	H···*A*	*D*···*A*	*D*—H···*A*
O13—H79···O12	0.81 (3)	1.83 (3)	2.632 (2)	171 (3)
O14—H80···O4	0.76 (3)	2.27 (3)	2.941 (2)	148 (3)
O15—H81···O4	0.82 (3)	1.79 (3)	2.583 (2)	160 (3)
O16—H82···O18	0.82 (3)	1.86 (3)	2.684 (3)	178 (4)
O17—H83···O8	0.82 (3)	1.99 (3)	2.783 (2)	165 (3)
O18—H84···O8	0.82 (4)	1.94 (4)	2.723 (3)	160 (4)

## References

[bb1] Anwander, R. (2002). *Applied Homogeneous Catalysis with Organometallic Compounds*, edited by B. Cornils & W. A. Herrmann, pp. 974–1013. Weinheim: Wiley-VCH.

[bb2] Atwood, D. A. (2016). *Sustainable Inorganic Chemistry.* New York: Wiley.

[bb3] Bruker (2016). *APEX2* and *SAINT*. Bruker AXS Inc., Madison, Wisconsin, USA.

[bb4] Bünzli, J. G. (2017). *Eur. J. Inorg. Chem.* pp. 5058–5063.

[bb5] Bünzli, J. G. & Piguet, C. (2005). *Chem. Soc. Rev.* **34**, 1048–1077. 10.1039/b406082m 16284671

[bb6] Chen, J. (2016). *Application of Ionic Liquids on Rare Earth Green Separation and Utilization.* Berlin, Heidelberg: Springer-Verlag.

[bb7] Friebe, L., Nuyken, O. & Obrecht, W. (2006). *Adv. Polym. Sci.* **204**, 1–154.

[bb8] Greenham, N. C., Samuel, I. D. W., Hayes, G. R., Phillips, R. T., Kessener, Y. A. R. R., Moratti, S. C., Holmes, A. B. & Friend, R. H. (1995). *Chem. Phys. Lett.* **241**, 89–96.

[bb9] Guillou, O., Daiguebonne, C., Calvez, G. & Bernot, K. (2016). *Acc. Chem. Res.* **49**, 844–856. 10.1021/acs.accounts.6b00058 27082821

[bb10] Kobayashi, S. & Anwander, R. (2001). *Lanthanides: Chemistry and Use in Organic Synthesis. Topics in Organometallic Chemistry*, Vol. 2, pp. 1–307. Berlin, Heidelberg: Springer-Verlag.

[bb11] Krause, L., Herbst-Irmer, R., Sheldrick, G. M. & Stalke, D. (2015). *J. Appl. Cryst.* **48**, 3–10. 10.1107/S1600576714022985 PMC4453166 26089746

[bb12] Mello, J. C. de, Wittmann, H. F. & Friend, R. H. (1997). *Adv. Mater.* **9**, 230–232.

[bb13] Minyaev, M. E., Korchagina, S. A., Tavtorkin, A. N., Churakov, A. V. & Nifant’ev, I. E. (2018*b*). *Acta Cryst.* C**74**, 673–682. 10.1107/S2053229618006666 29870002

[bb14] Minyaev, M. E., Korchagina, S. A., Tavtorkin, A. N., Kostitsyna, N. N., Churakov, A. V. & Nifant’ev, I. E. (2018*c*). *Struct. Chem.* **29**, 1475–1487. 10.1107/S2053229618006666 29870002

[bb15] Minyaev, M. E., Nifant’ev, I. E., Tavtorkin, A. N., Korchagina, S. A., Zeynalova, S. S., Ananyev, I. V. & Churakov, A. V. (2017). *Acta Cryst.* C**73**, 820–827. 10.1107/S2053229617012979 28978790

[bb16] Minyaev, M. E., Nifant’ev, I. E., Tavtorkin, A. N., Korchagina, S. A. & Zeynalova, S. S. (2015). *Acta Cryst.* E**71**, 443–446. 10.1107/S2056989015005563 PMC4420106 25995851

[bb17] Minyaev, M. E., Tavtorkin, A. N., Korchagina, S. A., Bondarenko, G. N., Churakov, A. V. & Nifant’ev, I. E. (2018*a*). *Acta Cryst.* C**74**, 590–598. 10.1107/S2053229618005570 29726468

[bb18] Nelyubina, Y. V., Puntus, L. N. & Lyssenko, K. A. (2014). *Chem. Eur. J.* **20**, 2860–2865. 10.1002/chem.201300566 24488449

[bb19] Nifant’ev, I. E., Tavtorkin, A. N., Korchagina, S. A., Gavrilenko, I. F., Glebova, N. N., Kostitsyna, N. N., Yakovlev, V. A., Bondarenko, G. N. & Filatova, M. P. (2014). *Appl. Catal. Gen.* **478**, 219–227.

[bb20] Nifant’ev, I. E., Tavtorkin, A. N., Shlyahtin, A. V., Korchagina, S. A., Gavrilenko, I. F., Glebova, N. N. & Churakov, A. V. (2013). *Dalton Trans.* **42**, 1223–1230. 10.1039/c2dt31505j 23138152

[bb21] Sheldrick, G. M. (2008). *Acta Cryst.* A**64**, 112–122. 10.1107/S0108767307043930 18156677

[bb22] Sheldrick, G. M. (2015*a*). *Acta Cryst.* C**71**, 3–8.

[bb23] Sheldrick, G. M. (2015*b*). *Acta Cryst.* A**71**, 3–8.

[bb24] Wrighton, M. S., Ginley, D. S. & Morse, D. L. (1974). *J. Phys. Chem.* **78**, 2229–2233.

[bb25] Zhang, Z., Cui, D., Wang, B., Liu, B. & Yang, Y. (2010). *Struct. Bond.* **137**, 49–108.

